# Molecular determinants for dsDNA translocation by the transcription-repair coupling and evolvability factor Mfd

**DOI:** 10.1038/s41467-020-17457-1

**Published:** 2020-07-27

**Authors:** Christiane Brugger, Cheng Zhang, Margaret M. Suhanovsky, David D. Kim, Amy N. Sinclair, Dmitry Lyumkis, Alexandra M. Deaconescu

**Affiliations:** 1 Department of Molecular Biology, Cell Biology and Biochemistry, Brown University, Providence, RI 02903 USA; 2 Laboratory of Genetics, The Salk Institute for Biological Studies, La Jolla, CA 92093 USA; 3 Department of Computational and Structural Biology, The Scripps Research Institute, La Jolla, CA 92093 USA

**Keywords:** DNA repair enzymes, Cryoelectron microscopy

## Abstract

Mfd couples transcription to nucleotide excision repair, and acts on RNA polymerases when elongation is impeded. Depending on impediment severity, this action results in either transcription termination or elongation rescue, which rely on ATP-dependent Mfd translocation on DNA. Due to its role in antibiotic resistance, Mfd is also emerging as a prime target for developing anti-evolution drugs. Here we report the structure of DNA-bound Mfd, which reveals large DNA-induced structural changes that are linked to the active site via ATPase motif VI. These changes relieve autoinhibitory contacts between the N- and C-termini and unmask UvrA recognition determinants. We also demonstrate that translocation relies on a threonine in motif Ic, widely conserved in translocases, and a family-specific histidine near motif IVa, reminiscent of the “arginine clamp” of RNA helicases. Thus, Mfd employs a mode of DNA recognition that at its core is common to ss/ds translocases that act on DNA or RNA.

## Abstract (web summary)

Transcription-repair coupling factors (TRCFs) are large ATPases that mediate the preferential repair of the transcribed DNA strand. Here the authors reveal the cryo-EM structure of DNA-bound Mfd, the bacterial TRCF, and provide molecular insights into its mode of action.

## Introduction

Given that DNA serves as a track for multiple essential molecular machines, including those carrying out replication, transcription, and repair, the movement of nucleic-acid translocases along single-stranded (ss) or double-stranded (ds) DNA is highly regulated spatiotemporally^1^. This control has a central role in development, neurodegeneration, aging, and the diseased state^2^, and despite advances, still remains insufficiently understood. Translocation on dsDNA has been challenging to study structurally because many DNA motors have little sequence specificity, are difficult to trap within a crystal, and translocation on dsDNA, as opposed to on ssDNA, leads to no detectable product. These challenges are compounded by the finding that translocases often function while coupled to macromolecular machines. They also display varied processivity and coupling to ATP hydrolysis, and while featuring conserved sequence motifs, have mechanisms of action that are modulated by accessory domains in family-specific ways, evading facile generalizations.

**Fig. 1:**
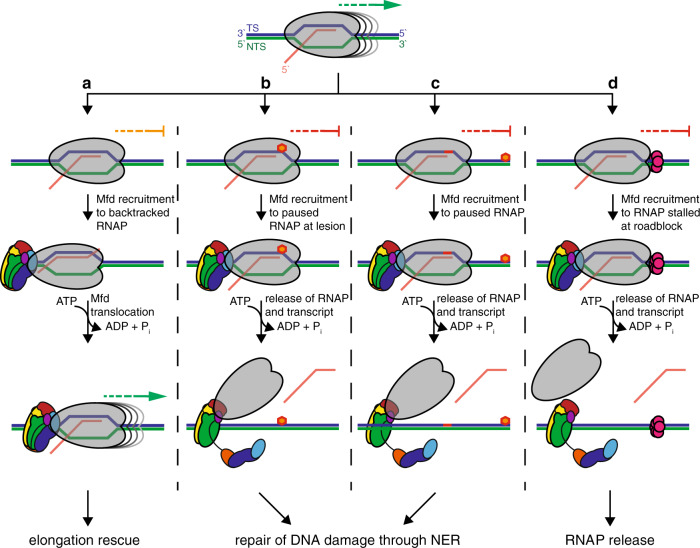
All Mfd functions are dependent on translocation on DNA or action upstream of transcription elongation complexes. Shown at the top is a schematic of an elongating RNAP (gray) with labeled nucleic-acid moieties (RNA, red; NTS, non-template strand, green; TS, template strand, blue) that can either become stalled or temporarily paused as indicated in **a**–**d**. **a** In Mfd-dependent rescue of class II transcriptional pausing, Mfd binds to backtracked RNAPs and promotes their forward translocation thereby rescuing transcript elongation^16,26^. **b** In canonical TCR, RNAP becomes stalled at a lesion (orange hexagon) on the TS and recruits Mfd (colored by domain, with D1a and D1b in blue, D2 in cyan, D3 in orange, D4 (RID) in magenta, D5 in yellow, D6 in green and D7 in red), either through 3D-diffusion or 1D-diffusion, enabled by a catch-up-then release mechanism dependent on ATP hydrolysis^16^, disruption of the D2–D7 clamp, RNA release, and UvrAB recruitment^29,31^. **c** In repair “at a distance”, Mfd is recruited to RNAP paused at class II pause signals (red line), which is first released off the nucleic-acid chains and acts as a processivity factor for Mfd to translocate toward downstream lesions in the TS, and initiate strand-specific repair^17^. **d** When RNAPs collides with protein roadblocks head-on, including replisomes, Mfd will release RNAP off the nucleic-acid chains, thereby freeing the DNA for other DNA-based processes^18,19^.

Transcription-repair coupling factors (TRCFs) are large superfamily 2 ATPases that mediate the preferential repair of the transcribed DNA strand (aka transcription-coupled DNA repair, TCR) in organisms ranging from bacteria to humans^3–5^. These factors provide a complex but poorly understood example of regulated translocation on dsDNA. At its core, TCR appears universally conserved, and relies on (1) the ability of transcription-repair coupling ATPases to release damage-stalled RNA polymerases (RNAPs) off the nucleic-acid template owing to ATP-driven translocation on dsDNA upstream of the transcription bubble, and (2) on their ability to recruit nucleotide excision repair (NER) machinery^6^. In bacteria, a single protein, Mfd (aka TRCF) is necessary and sufficient for the coupling process^7,8^. Although additional pathways have been implicated in TCR^9^, Mfd remains the only bacterial factor for which both RNAP release and repair enzyme recruitment functions have been demonstrated^3,8,10–14^. Critically, Mfd associates with RNAP in cells even in the absence of exogenous DNA damage^15^, it decreases class II transcriptional pausing^16^, promotes strand-specific repair “at a distance” downstream of a transcriptional pause site^17^, and dissociates transcription elongation complexes (TECs) stalled not only by DNA damage, but also by protein roadblocks^18^, including replication forks colliding head-on with the transcription machinery^19^. Thus, given these different contexts in which it acts on TECs (Fig. 1), Mfd is more appropriately viewed as a general transcription factor. Paradoxically, under certain conditions, Mfd acts as an evolvability factor, promoting hypermutation, the accelerated evolution of lagging-strand genes^20,21^ and the rapid development of resistance to multiple, unrelated classes of antibiotics^22–25^. This makes Mfd an attractive target for the development of a broad-spectrum anti-evolution drug, which could be administered in combination with well-characterized antibiotics to curtail the worldwide crisis of antimicrobial resistance.

**Fig. 2:**
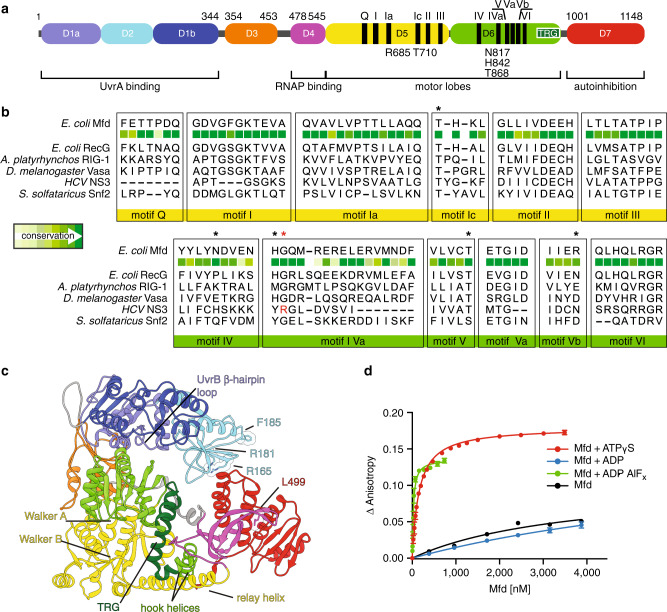
DNA binding by Mfd becomes tightest in the presence of transition state analog ADP*•*AlF_x_. **a** Domain organization of *E. coli* Mfd with annotated conserved sequence motifs and key residues mutated in this study. **b** Structure-based sequence alignment of the ATPase motifs of Mfd and other dsDNA and ssDNA translocases used for structural modeling purposes. Mutated residues are indicated by asterisks and sequence conservation, based on an alignment of Mfd proteins is color-coded from dark green (strongly conserved) to light green (variable). ATPase domain motifs are shown underneath for *Escherichia coli* RecG, *Sulfolobus solfataricus* Swi2/Snf2, *Drosophila melanogaster* Vasa, *HCV* NS3, and *Anas platyrhynchos* RIG-1. The arginine clamp R393 of *HCV* NS3 is shown in red. **c** Structural overview of nucleotide-free *E. coli* Mfd (PDB ID 2EYQ) colored by domain and with annotated ATPase motifs and key structural elements. Shown as CPK models are residues implicated in UvrA and RNAP binding^29,31^. **d** Fluorescence anisotropy DNA-binding curves for Mfd in distinct nucleotide states: nucleotide-free (black), with 2 mm ATPγS (red), ADP (blue), or ADP•AlF_x_ (green). Error bars represent S.D.M. (*n* = 3) and are often smaller than symbols. Curves obtained in the absence/presence of ATPγS and ADP are replotted for convenience from the study by Le et al.^16^ Source data are provided as a Data Source file.

Mfd translocation on dsDNA is central to all Mfd functions. This is an ATP-dependent^13,26–29^ and regulatable process^16^, which it shares with chromatin remodelers belonging to the same superfamily 2 (SF2) of ATPases. Like chromatin remodelers, Mfd binds and remodels a large macromolecular assembly—the TEC, composed of core RNAP, DNA scaffold, and nascent RNA. TECs activate the Mfd translocase to a level at which processive translocation can readily be detected using classic biochemistry^26,30^. The structural changes underlying this activation are likely complex and may involve multiple steps^27^ and interlocked structural elements^29,31^. Mfd is composed of an ATP-dependent motor core composed of domains D5 and D6 as well as six ancillary domains, including D2 and D7 that pack against each other as an inhibitory “clamp” to restrain the ATPase (Fig. 2a–c) and mask binding determinants for recruitment of UvrA, a component of early NER^29,31^. Conformational changes in full-length *Escherichia coli* Mfd occurring during its functional cycle have remained speculative since only a single structure—that of nucleotide-free *E. coli* Mfd^31^—has been reported in peer-reviewed literature. Clamp opening was originally proposed to be prerequisite for translocation and dependent on the interaction with RNAP^30^ (Fig. 1). Recent and more-sensitive single-molecule studies have demonstrated that in fact Mfd can also translocate on naked DNA, albeit with limited processivity^16^, suggesting that Mfd exists in a dynamic conformational equilibrium that can be shifted by TEC binding. However, provided that ATP is supplied, Mfd can make excursions to the translocation-competent form even in the absence of RNAP^16^, suggesting that Mfd locates its targets not only by 3D diffusion, but also a more efficient 1-D search along dsDNA, possibly colliding with stalled/paused TECs ahead of it^16^. This scenario can lead to two outcomes: RNAP rescue by forward translocation (Fig. 1a) or dissociation (Fig. 1b–d), occurring when RNAP encounters severe hindrances to forward movement^16^. It is important to note that Mfd processivity on naked DNA is substantially lower than that of Mfd bound to a RNAP that has been released off the DNA chains, but remains associated with Mfd via interactions with domain D4, and possibly other unknown binding sites^16^.

Despite intense research in the last decade, the mechanochemistry of TRCFs remains poorly defined. How do TRCFs recognize DNA and what are the conformational changes associated with translocation on dsDNA? Here, we shed light on these questions by defining the path of DNA across Mfd using electron cryo-microscopy (cryo-EM), structure-guided mutagenesis, and functional assays. We demonstrate that in the absence of RNAP, independent, single substitutions within the two lobes of the Mfd motor core lead to severe DNA-binding defects, and that translocation relies on the existence of a moderate and a strong binding state that correspond to the ATP- and ADP•AlF_x_-bound states, respectively. We pinpoint conserved residues within translocase motifs Ic, IV, V, and Vb as contributing to DNA loading, and identify an evolutionary conserved threonine in motif Ic and a family-specific histidine near motif IVa as critical for translocation. We also show that DNA binding to Mfd in the presence of transition state analog ADP•AlF_x_ leads to large-scale rotation of the UvrB homology module, resulting in disruption of the D2–D7 clamp as well as repositioning of domain D3 of hitherto unknown function. This leads to the breaking of contacts of D3 with D5 and D6 and the establishment of new contacts with D7. Our study provides the first glimpse of a substrate-bound bacterial TRCF, and suggests that these ancient ATPases utilize a mode of DNA recognition that at its core, is common to ss/ds nucleic-acid translocases.

## Results

### Mfd binds dsDNA tightly with transition state analog ADP•AlF_x_

For processive autonomous movement on dsDNA (i.e., not dependent on tethering to RNAP) to occur, Mfd must cycle through several states, and must possess at least two DNA contact points, utilized differentially during the ATPase cycle. Mfd binding to DNA with non-hydrolysable ground ATP mimics, ATPγS and AMPPNP has been well documented^14,32^. ATP hydrolysis leads to DNA dissociation, e.g., Mfd translocation towards the end of the fragment^3,26,33^.

Importantly, although ATP binding is thought to reposition D5/D6 and align the ATPase motifs for catalysis^31^, there are no reports of a second state tightly associating with DNA. This would be critical because it would provide the necessary powerstroke for unidirectional movement. We have thus asked whether trapping such an alternate state might be possible using ADP and ADP•AlF_x_. During ATP hydrolysis, the γ-phosphate of ATP passes through a trigonal planar configuration with three equatorial oxygen atoms and two axial ligands, an oxygen from the β-phosphate and the attacking water molecule. To mimic this state, we used Mg^2+^•ADP•AlF_x_, the most extensively employed analog of the ATP hydrolysis transition state. This binds as an AlF_4_^−^ species to most ATPases and adopts an octahedral geometry when complexed to the β-phosphate and the nucleophilic water moiety^34^. To quantify DNA binding under equilibrium conditions, we used a fluorescence anisotropy assay with a hexachlorofluorescein (HEX)-labeled 40mer dsDNA fragment. In the presence of ATPγS, the equilibrium dissociation constant, *K*_d_, was 151 ± 3 nm (Fig. 2d, Supplementary Table 1), in agreement with previous determinations^29^. We detected much reduced binding with ADP and enhanced binding with ADP•AlF_x_ (*K*_d_^ADP•AlFx^ = 24 ± 2 nm). In agreement with several reports in *E. coli*^29^, but inconsistent with reports of oligomeric *Mycobacterium tuberculosis* Mfd^35^, we observed no oligomerization by gel filtration (Supplementary Fig. 1a), suggesting that the increase in affinity in the presence of the transition state is owing to intramolecular rearrangements rather than oligomerization, as in other SF2 nucleic-acid translocases^36^.

### The primary DNA-binding site in Mfd is its motor core

Both our study and previous work have not detected stable binding of Mfd to dsDNA in the absence of nucleotide^14^. Modeling of a duplex DNA-Mfd complex based on similarity with other SF2 proteins such as *Sulfolobus solfataricus* Snf2 is consistent with this finding. In the nucleotide-free form, the motor core of Mfd is more open. dsDNA modeled onto nucleotide-free Mfd contacts a highly conserved and positively charged D5 surface patch centered around invariant K712, but is nevertheless positioned too far away from D6 for direct contacts with this domain (Supplementary Fig. 1b–d), supporting that affinity is dictated by nucleotide status and suggesting that D6 swings down to clamp the DNA (Supplementary Fig. 1b–d). However, the conformation of the ATPase motor module in many translocases/helicases, and by extension, in Mfd, is modulated not only by nucleotide status but also interactions with family-specific accessory domains^37^, suggesting that changes that are more global rather than restricted to the motor module.

**Fig. 3:**
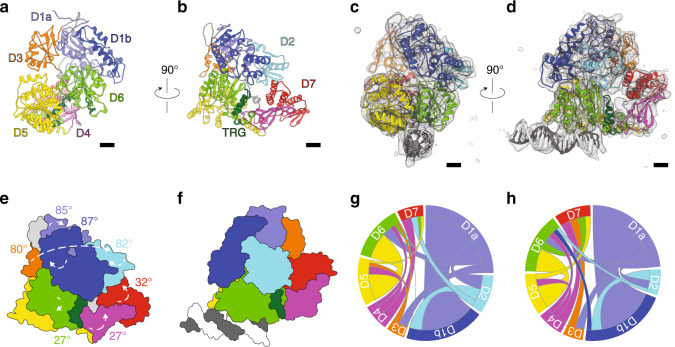
Cryo-EM reconstruction of dsDNA-bound Mfd reveals a mobile D3 module and the interaction of dsDNA with the motor lobes. **a**, **b** Orthogonal views of nucleotide-free Mfd (PDB ID 2EYQ). Scale bar is equivalent to 10 Å. **c**, **d** Orthogonal views of the cryo-EM reconstruction (gray surface) with the fitted Mfd model shown as a cartoon and colored as in a. DNA is shown in gray. **e**, **f** Cartoons of Mfd structures in the nucleotide free **e** and DNA-bound form **f** after superposition on the Cα trace of D5 and highlighting the relative rotation of Mfd domains in the free and tight DNA-bound state. **g**, **h** Chord plots highlighting intramolecular rearrangements in Mfd before **g** and after **h** DNA binding. In all panels, Mfd is colored by domain as in Fig. 2a.

Our observation that ADP•AlFx enhances the affinity for DNA allowed us to reconstitute stable Mfd-DNA complexes, and prepare specimens suitable for single-particle cryo-EM for structure determination. For our DNA substrate, we chose a 21mer blunt dsDNA fragment, consistent with previously determined Mfd footprints of ~26bp^16,26,29^. This allowed us to restrict binding to one Mfd molecule per DNA fragment. Initial reconstructions obtained from untilted specimens were severely affected by the preferred orientation of the particles at the air-water interface, limiting resolution. We thus acquired images at 30° tilts, which allowed us to obtain a more isotropic map resolved to 5.5 Å (Methods, Supplementary Fig. 2, and Supplementary Table 2). This enabled us to readily dock all the individual domains of Mfd^31^ into the map and perform real-space refinement to obtain the final model. At this resolution, we do not observe density that can be assigned to the transition state analog moiety. The model (Fig. 3) affords several critical observations.

First, our reconstruction reveals an unexpected large-scale swiveling motion of the UvrB homology module (D1a-D2-D1b), which results in a ~49 Å displacement and an 82° rotation of D2. Importantly, D1a-D2-D1b appears to move in concert, as a rigid body, which is consistent with previous structures of D1aD2D1b and D1aD2D1bD3 truncations, which assumed the same structure in isolation as in the context of the full-length, nucleotide-free protein (r.m.s.d. of 0.8 Å between PDB ID 2EYQ and PDB ID 2B2N and 0.6 Å between PDB ID 2EYQ and PDB ID 3HJH)^32,38^. Limited proteolysis of Mfd in the absence/presence of nucleotides and Mg•ADP•AlF_x_ did not reveal differences in protease susceptibility (Supplementary Fig. 3), suggesting that the conformational changes we observe are induced by DNA binding and not by the progression from the ATP ground to the transition state. Recruitment of UvrA might thus preferentially occur in the ADP-Pi state and might promote product release and dissociation of Mfd off the DNA, leaving behind an excinuclease loaded in a strand-specific manner.

**Fig. 4:**
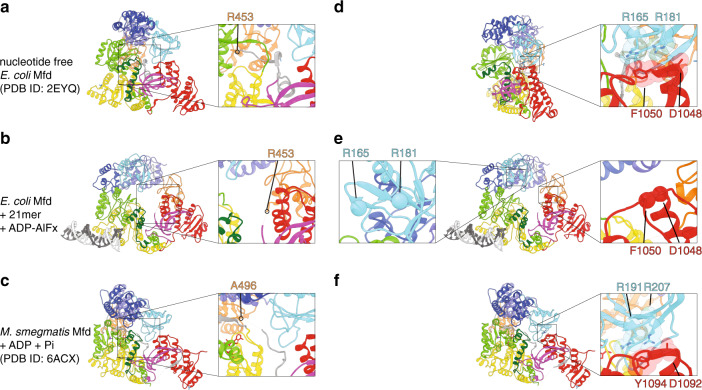
DNA binding unmasks the UvrA recruitment surface within domain D2 of Mfd. **a**–**c** Top view of nucleotide-free *E. coli* Mfd **a**, *E. coli* Mfd complexed to DNA and ADP-AlF_x_ **b** and *M. smegmatis* Mfd bound to ADP and Pi **c** (PDB ID 6AXC). Insets show the D3–D4 linker, and C-terminal residues in D3 as positional markers. The D3–D4 linker is not visible in our map of DNA-bound Mfd **b** or in *M. smegmatis* Mfd **c**. **d**–**f** Overall views of Mfd with insets highlighting the disruption of the D2–D7 interaction upon DNA binding. Amino acids involved in domain–domain interactions (R165, R181, D1048, F1050) are shown as CPK with stick models. Middle inset highlights the unmasking of R165 and R181 (spheres), important for UvrA recruitment. In all panels, Mfd is colored by domain as in Fig. 2a.

Second, particularly striking is the repositioning of domain of unknown function D3, which swings from the ventral side laterally toward D7 upon DNA binding (Fig. 3 and Supplementary Movie 1), establishing new bridging interactions with the UvrB homology module and D7. This represents a large scale ~47 Å displacement of D3 enabled by its flanking flexible linkers connecting D3 to D1b and D4 (Figs. 2a–b, 3). The distance between the termini of the fitted D3 and D4 (36 Å) is fully compatible with the length of this partially disordered D3–D4 linker. Consistent with our data, the sequence corresponding to D3 in the structure of D1aD2D1bD3 was completely disordered^32^, pointing to it forming a dynamic and separate structural unit. Recent structures of mycobacterial Mfd in the presence of ADP and ADP•Pi also revealed movement of D3^39^, but this is distinct from what we observe. In mycobacterial Mfd, sharing ~33% identity with *E. coli* Mfd, D3 is repositioned when ADP and Pi are bound such that it contacts D1a and D5 (Fig. 4a–c). These contacts are presumably important for nucleotide binding, as they are accompanied by a flip-flop action of the D3–D4 linker (Fig. 4 and Supplemental Fig. 5)^39^. Movement of this linker relieves steric occlusion of the conserved phenylalanine of motif Q (F641 mycobacterial Mfd, F599 in *Eco* Mfd), which becomes free to stack against the adenine base of ADP. In contrast, when Mfd is bound to DNA, D3 makes contacts with D1a and D7 (Fig. 3), swapping position with D2. The UvrA-binding surface on D2, including residues critical for binding such as R165 and R181^29^ (Fig. 2d–f) becomes fully exposed. Contacts of D2 with conserved residues in D7, D0148, and F1050 are lost.

**Fig. 5:**
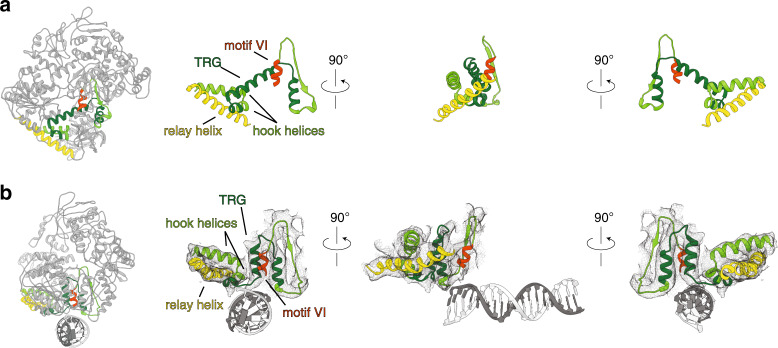
Structural interlocking of the TRG motif, motif VI, and relay helix. **a** View of motif VI (red), TRG motif (dark green), hook helices (green), and relay helix (yellow) in the context of full-length nucleotide-free *E. coli* Mfd (left) and, for emphasis, as close-ups. **b** Similar views of DNA-bound *E. coli* Mfd highlighting conformational changes in this region The TRG helical hairpin closes and a bend is introduced into the relay helix.

Last, but not least, our reconstruction confirms the long-hypothesized role of the TRG motif in DNA translocation. The TRG (translocation in RecG) motif is common to both the RecG and Mfd subfamilies, and consists of a helical hairpin engaging a helical “hook” C-terminal to it (Fig. 2c). This hook was partially disordered in substrate-bound RecG, but helical in Mfd and wrapped around yet another helix—the relay helix, connecting D4 to the motor core (Fig. 2c). In our reconstruction, the two helices of the TRG snap together and are likely stabilized by ADP•AlF_x_. binding. In contrast, in nucleotide-free Mfd, the TRG helices are splayed open by motif VI helix ATP sensor^31^. The closed TRG motif engages part of the hook structure but releases motif VI, which undergoes a 46 Å displacement upon rotation of D6, and indirectly modulates the conformation of the relay helix, which becomes slightly bent and rotates about a hinge located in the loop connecting it to D5 (Fig. 5 and Supplementary Movie 1). Thus, we conclude that domain D3 of hitherto unknown function plays a critical role in unmasking of the UvrA binding site and that the interlocking of motif VI, TRG, hook and relay helices enable the complex and long-range conformational changes associated with DNA binding.

### Linchpin residues for DNA loading span both lobes of the motor core

For translocation to occur, Mfd must first load the DNA. Our reconstruction suggests that this involves D5 and D6 exclusively, and no other contacts (Fig. 3a–f), although secondary binding determinants might be unmasked under very specific conditions.

We have thus asked if isolated Mfd domains might contribute to ss or dsDNA binding. We have purified Mfd functional modules as defined by the crystal structure of *E. coli* Mfd (UvrB homology module Mfd^D1aD2D1b^, domain of unknown function Mfd^D3^, RNAP interaction domain Mfd^D4^, and clamp domain Mfd^D7^ (Fig. 2a and Supplementary Fig. 4) with the exception of Mfd^D5D6^. We then assessed DNA binding with either a 40mer HEX-labeled dsDNA or a shorter Cy3-labeled double- and single-stranded 27mer. As expected, we observed no stable binding to any of the purified modules (Supplementary Fig. 4). However, we cannot rule out lower affinity, secondary binding sites that might require initial contacts with Mfd^D5D6^, a DNA fragment longer than what was used for our EM reconstruction, or a particular DNA structure, akin to an open or partially open bubble. On longer cellular DNA substrates, multiple domains may be required for DNA binding and contacts with individual modules, as defined here, may be too weak to be detected.

**Fig. 6:**
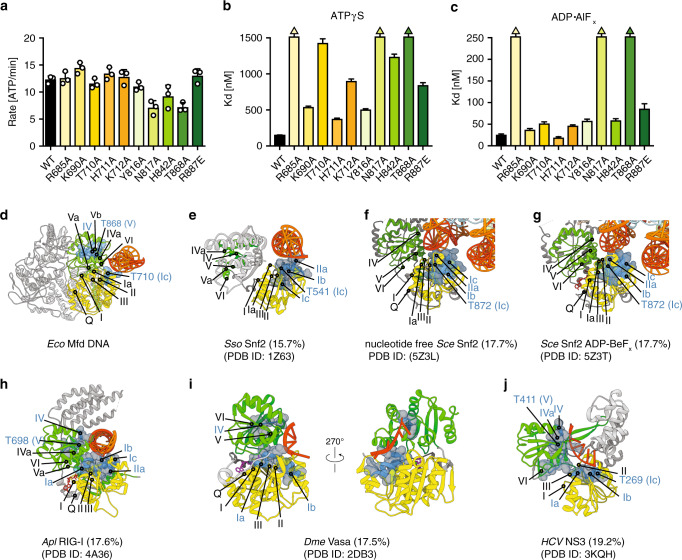
Key roles for R685, N817, and R868 in DNA loading. **a** Steady-state ATPase activities of single Mfd variants. ATP hydrolysis rates were measured using an ATP/NADH-coupled ATPase assay. Data shown are the means ± SD (*n* = 3). Variants are color-coded by domain as in Fig. 2a (D5 variants, yellow hues; D6 variants, green hues). Data for Mfd^R685A^ and Mfd^N817A^ are replotted for convenience from Le et al.^16^. **b**–**c** Equilibrium dissociation constants for the Mfd-dsDNA interaction derived from fluorescence anisotropy binding curves. D5 and D6 variants are color-coded by domain as in Fig. 2a. Data represent mean values ± SD (*n* = 3). Error bars are often smaller than symbols. Arrows indicate data sets that could not be reliably fit to a binding model owing to compromised affinity for DNA, and likely represent an overestimation of the true affinity. Binding curves are shown in Supplementary Fig. 7. **d**–**j** Structural comparison between double-stranded nucleic-acid translocases and *Escherichia coli* (*Eco*) Mfd (d) using superpositions restricted to the Cα trace of D5. Shown are *Sulfolobus solfataricus* (*Sso*) Snf2 (r.m.s.d. of 1.1 Å, **e**), *Saccharomyces cerevisiae* (*Sce*) Snf2 in its nucleotide-free (r.m.s.d of 1.4 Å, **f**) and ADP•BeF_x_ -bound state (r.m.s.d. of 1.4, **g**), RIG-1 (r.m.s.d. of 1.0 Å, **h**) and ssRNA translocases, *Drosophila melanogaster* (*Dme*) Vasa (r.m.s.d. of 0.9 Å, **i**) and *HCV* NS3 (r.m.s.d. of 1.1 Å, **j**). Bound Mg^2+^ is shown as a dark green sphere, analog ADP•AlF_3_^−^ as an aquamarine CPK model, bound nucleotide as magenta sticks, ADP as a red CPK model, BeF_x_ as a dark red CPK model and nucleic-acid strands are colored red and orange. The two lobes of the ATPase core are in yellow (N-terminal lobe) and green (C-terminal lobe), except for DNA-binding residues, which are highlighted as slate CPK models. Other domains are shown as a gray ribbon with annotated ATPase motifs. Those motifs contacting DNA are color-coded in slate font and some are not visible in these views. Indicated in parenthesis is the degree of conservation between the various motor cores and D5 and D6 of *E. coli* Mfd. Source data are provided as a Source Data file.

Although the EM reconstruction allowed us to identify residues proximal to DNA, specific contacts with the nucleic acid are not resolved with confidence at this resolution. To identify residues critical for DNA loading in both the ATP- and ADP•AlF_x_ states, we used our EM reconstruction but also took advantage of evolutionary relationships and structural superpositions with well-characterized nucleic acid bound ATPases. Structural alignments with RecG, the helicase most closely related to Mfd, proved unfruitful, as crystallized RecG only contains a short fork DNA that does not reach across the helicase domains^40^. Instead, we extended our analyses to other ss/ds nucleic-acid translocases of known structure. We focused on the similarity with Snf2, an SF2 chromatin remodeler for which several structural models in different functional states are available. These structures include models of Snf2 bound to dsDNA^41^, akin to the DNA moiety found in our specimen, but also curved DNA, as found in the structure of Snf2 bound to nucleosomes^42,43^. We combined the information derived from our EM reconstruction and these superpositions (Supplementary Figure 6), and selected a set of well-conserved residues, highlighted in Fig. 6a and the Supplementary Movie 1, as likely key for DNA recognition. We constructed Mfd variants carrying substitutions in either D5 (Mfd^R685A^, Mfd^K690A^, Mfd^T710A^, Mfd^H711^, Mfd^K712A^) or D6 (Mfd^Y816A^, Mfd^N817A^, Mfd^H842A^, Mfd^T868A^, Mfd^R887E^) (Fig. 2a). All were active in ATPase assays at levels comparable to wild type (Fig. 6a), indicating they are competent for nucleotide binding and hydrolysis, and consistent with proper folding, which was assessed using circular dichroism (Supplementary Fig. 7b). We then assessed binding to the same 40mer HEX-labeled dsDNA used above. The variants displayed various degrees of DNA-binding defects in the presence of both ATPγS (Fig. 6b, Supplementary Fig. 7c and Supplementary Table 1) and ADP•AlF_x_ (Fig. 6c, Supplementary Fig. 7d and Supplementary Table 1). Interactions with DNA were more sensitive to salt in the ATPγS than the ADP•AlF_x_ state (Supplementary Fig. 7e, f), pointing to the formation of non-electrostatic contacts as partially accounting for the gain in affinity in the transition state.

Mfd^R685A^, Mfd^N817A^, and Mfd^T868A^ were severely impaired with both ATPγS and ADP•AlF_x_, indicating a critical role in loading DNA. T868 is particularly intriguing, as it is located in conserved helicase motif V (Fig. 2a–b), which in several cases has been shown to contribute to either ATP or oligonucleotide binding^44^. In the case of Mfd, substitution of T868 affects DNA binding severely, and less so ATP binding or hydrolysis (Fig. 6a–c). This is not without precedent. The structure of duck RIG-I RNA helicase bound to 19mer dsRNA and ADP•AlF_x_, shows this highly conserved threonine (T698 in duck RIG-I) in direct contact with a phosphate of the RNA backbone (Fig. 6h), making a hydrogen bond with the phosphoryl oxygen atom^45^. However, mutation of this conserved residue to alanine in human RIG-I eliminates dsRNA binding while retaining ATP hydrolysis activity^46^, suggesting that this residue confers affinity that is uncoupled from the splitting of the phosphoanhydride bond. In *Thermotoga maritima* RecG, the mutation of T478 reduces fork reversal (i.e., translocation) without affecting ATP hydrolysis^47^. A structurally equivalent threonine, T546, also contacts the RNA phosphate backbone in the structure of Vasa RNA helicase bound to ssRNA and AMPPNP (PDB ID 2DB3, Fig. 6i)^48^. At last, the SF2 helicase domain of virally encoded DExH helicase NS3 also has a similar conserved threonine, and this was seen to contact oligonucleotide in multiple structural studies^49,50^. As with Mfd, mutation of this threonine (T411 in NS3h, Fig. 6j) does not affect its basal ATPase activity, but abolishes oligonucleotide binding and duplex unwinding activity^51^.

### Mfd mechanism of translocation is reminiscent of that used by RNA helicases

**Fig. 7:**
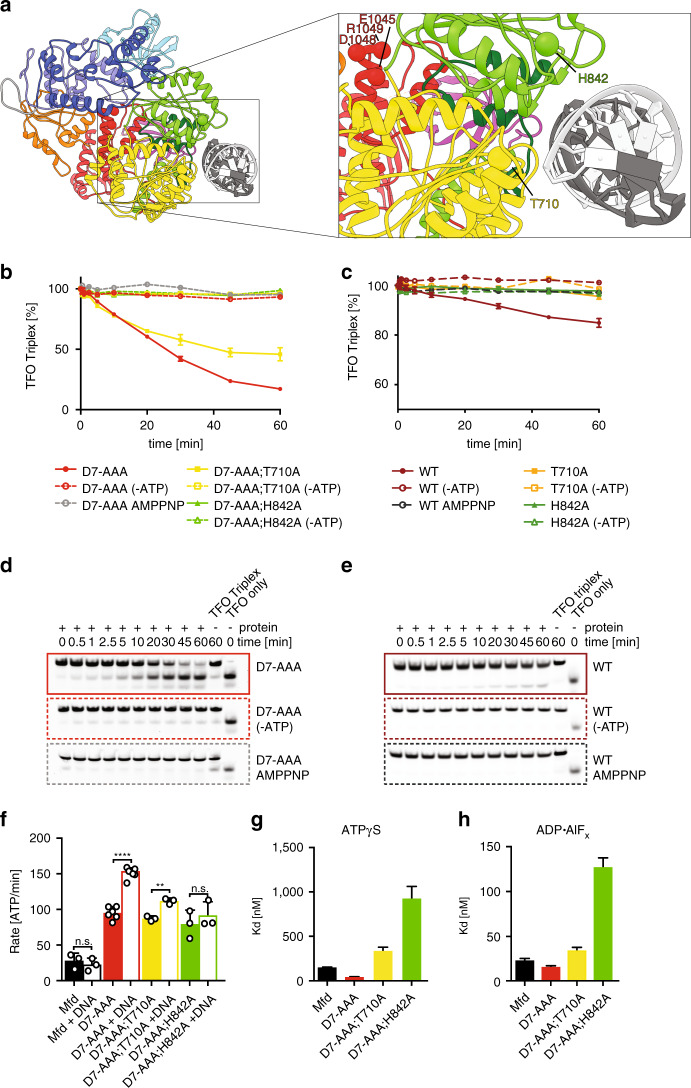
T710 and H842 are critical regulators of translocation by Mfd. **a** EM-based model of DNA-bound Mfd with boxed region highlighting the structural environment of T710 and H842 residues. The resolution of our reconstruction precludes precise determination of the conformation of motif IVa loop and of amino-acid rotameric forms. Crucial residues are indicated as Cα spheres, H842 in green, T710A in yellow and E1045, D1048, and R1049, mutated in Mfd D7^AAA^ in red. **b**–**c** TFO displacement assays carried out with Mfd^D7−AAA^ **b** and Mfd **c** variants. Percentage of remaining TFO-Triplex is plotted vs time to show translocation of Mfd variants. Plot shows means ± SD (*n* = 3), with error bars often smaller than symbols. **d**–**e** Electrophoretic separation of TFO displacement assays carried out with Mfd^D7−AAA^. Representative gels for Mfd^D7−AAA^ **d** and Mfd **e** +/-ATP and +AMPPNP are shown. Example gels for Mfd, Mfd^D7−AAA;T710A^ and Mfd^D7AAA;H842A^ variants are shown in Supplementary Fig. 8 (*n* = 3). **f** Steady-state ATPase activities of Mfd variants normalized to wild-type Mfd activity. Data represent means ± SD (*n* ≥ 3). **denotes *p* < 0.01, ****p < 0.0001 and ns, *p* > 0.05 (unpaired two-tailed *t* test). The *p* value for Mfd^D7AAA;T710^ with/without DNA was 0.0012. **g**, **h** Equilibrium dissociation constants for the Mfd-dsDNA interaction derived from fluorescence anisotropy binding curves obtained by titrating increasing amounts of protein into a mixture of 10 nm HEX-labeled 40mer DNA and 2.0 mm ATPγS **e** and ADP•AlF_x_. Data represent mean values ± SD (*n* = 3). *K*_D_ values are tabulated in Supplementary Table 1. Source data are provided as a Source Data file.

In the presence of ADP•AlF_x_, some of our variants (Mfd^K690A^, Mfd^T710A^, Mfd^K712A^, Mfd^Y816A^, Mfd^H842A^) showed only a slight impairment in function, or no DNA-binding defect at all, as was the case for Mfd^H711A^ (Fig. 6 and Supplementary Table 1). Most striking are the substitutions of T710A and H842A (Fig. 6a), which lead to severe loss of affinity in the ATPγS state, but had a comparatively minor effect on DNA affinity in the presence of ADP•AlF_x_. Using optical trapping, we have previously shown that in the context of a nucleotide-starved TEC, Mfd binds to the TEC such that D5 is located in front (adjacent to the TEC), followed by D6, and that upon ATPγS binding D6 moves 12 bp toward a stationary D5, approaching the RNAP footprint^16^. As ATP hydrolysis is required for processive translocation, this suggested to us that upon hydrolysis and/or product release, D5 may invade the RNAP footprint. We thus reasoned that the residues with nucleotide-dependent roles might be critical for cycling through all the states required for translocation. To probe translocation, we developed a fluorescent triplex displacement assay, based on the ability of dsDNA translocases to displace a fluorescently labeled triplex forming oligonucleotide (TFO) off a DNA duplex. Triplex displacement assays have been well established for constitutively hyperactive Mfd variants, such as Mfd^D7−AAA^, carrying the E1045A, D1048A, and R1049A substitutions in D7 to disrupt the inhibitory clamp interaction (Fig. 7a)^52^. Given the recently determined short processivity of Mfd of ~200 bp^16^, we employed a 70 bp substrate, shorter than the Mfd processivity length, in conjunction with the hyperactive Mfd^D7−AAA^ variant. We included in our reaction non-labeled ssDNA, for which Mfd has a weaker affinity^14^, but acts as an efficient trap to inhibit a contaminating endonuclease activity that initially severely reduced the signal corresponding to full-length TFO (not shown). In the presence of Mfd^D7−AAA^, free TFO accumulated over time, and this activity was inhibited by non-hydrolysable analog, AMPPNP and the absence of nucleotide (Fig. 7b–c and Supplementary Fig. 8). Both mutants, Mfd^D7−AAA;T710A^ and particularly Mfd^D7−AAA;H842A^ displayed defects in translocation and loading, but not ATP hydrolysis (Fig. 7f), and featured circular dichroism spectra similar to Mfd^D7−AAA^ (Supplementary Fig. 8b). The loading defects of these complex variants are somewhat different than the defects on the single T710A and H842A variants in the wild-type background (Figs. 6a–c, 7), and suggest that artificial disruption of the clamp interaction partially uncouples DNA binding from nucleotide status, consistent with a now derepressed enzyme^30^. As such, these variants display severe translocation defects, with Mfd^H842A^ and Mfd^T710^ displaying no detectable translocation activity (Fig. 7b, c). At higher protein concentrations used in an attempt to overcome the loss in affinity of the D7-AAA mutants, both in the presence of ATPγS and ADP•AlF_x_, the apparent translocation activity of both Mfd^D7−AAA^ and its two variants decreases (Supplementary Fig. 8), likely owing to multiple loading molecules interfering with translocation, which complicates the analysis. Interestingly, although wild-type Mfd is not stimulated by the addition of herring sperm DNA, Mfd^D7−AAA^ and to a lesser extent Mfd^D7−AAA;T710A^, and Mfd^D7−AAA;H842^ show an increase in ATPase activity (Fig. 7f and Supplementary Fig. 8f). Thus, whereas deregulation via clamp disruption hyperactivates Mfd, mutations within motifs Ic and IVa may repress Mfd activity.

H842 maps to a loop N-terminal to motif IVa and is conserved within the Mfd family, but not across translocases (Fig. 2a). However, based on a structural alignment, it is located close to where R393 in HCV NS3 maps (Supplementary Fig. 9). This has been coined the arginine clamp and is key for nucleic-acid binding and translocation^53^. In agreement with our findings, a set of structures of HCV translocase NS3h bound to DNA alone and in complex with ground-state ATP and transition state mimics revealed contacts of nucleic acid with R393 as well as a series of nucleotide-dependent structural transitions involving the equivalent of T710 (T269 in NS3h, Fig. 6j) as well as NS3h T411, the equivalent of T868^50^. T269 is in helicase motif Ic (aka TxGx motif), which while not originally identified as a conserved sequence, has been shown to be involved in nucleotide-dependent oligonucleotide binding in a variety of helicases, including NS3^44,54^. The NS3 structure obtained in the absence of ATP shows these threonine residues three nucleotides apart, however they are only two nucleotides apart in the structure obtained with the ATP transition state analog (Supplementary Fig. 9b–d)^49,50^. T411 maintains contact with the same nucleotide throughout the ATP hydrolysis cycle, whereas T269 releases the DNA in the ATP-bound state and makes a new contact with the DNA one nucleotide away when NS3h is in complex with the ATP transition-state analog. Conservation of these two threonine residues in Mfd, the nucleotide-dependent role of T710 and the confirmation that T868 is critical for DNA binding by Mfd regardless of the status of the phosphoanhydride bond point to common features of translocation in NS3h and Mfd, despite Mfd lacking helicase activity and binding preferentially to DNA rather than RNA.

## Discussion

Mfd-dependent processes rely heavily on its translocation on dsDNA. This is important not only for early events required to locate RNAP targets on the chromosome, but also for destabilizing TECs halted by DNA damage, pause signals, or various roadblocks^6^. Mfd action on transcription complexes effectively builds up torque to reanneal the upstream edge of the transcription bubble, shorten the bubble, and release the RNA transcript^26,55^. As a first step in shedding light on these events, here we describe the first structure of DNA-bound *E. coli* Mfd as well as complementary functional analyses. Our data allow for the first model for how nucleotide status and DNA binding control the breaking and formation of interdomain contacts and also reveal the molecular basis for DNA recognition by Mfd proteins.

We observe that DNA is bound by the two lobes of the motor core, consistent with a previous truncation analysis, which determined that in the presence of ATPγS, the DNA interaction determinants are restricted to D5 and most of D6^14^. In this study, domain boundary definitions were not based on structural information, and notably truncated ATPase motif VI and the TRG motif (Fig. 2a–c), with crucial roles in coupling nucleotide status to translocation^33^. However, even with our structure-based truncations, we detect no binding of ds/ssDNA to domains flanking the motor core. Previous studies noted a striking similarity between D1aD2D1b and a region in UvrB that contacts DNA during NER, and also locally melts it for damage recognition using a highly conserved beta hairpin at the interface between D1a and D1b^38^. This beta hairpin extension is absent in Mfd, is replaced by a short loop, which lacks the aromatics involved in nucleotide flipping in UvrB. This loop is located ~50 Å away from the D5/D6 binding site (Fig. 2c), further supporting that the similarity might be strictly architectural. Our reconstruction suggests that on DNA substrates longer than used here, the DNA fragment would likely approach the C-terminal region of the relay helix, where previous studies have identified one substitution (Mfd^W550A^) that enhances DNA binding and motor activity^56^. This could involve bending of the DNA across the protein surface, and/or modest rearrangements of the hook and TRG motifs, located in close proximity to the DNA.

We thus conceptualize DNA binding by Mfd in terms of two clusters of residues located at the tip of the D5 and D6 pincers that establish contacts with the substrate, primarily the tracking strand, and cycle between a high-affinity state (nucleotide-bound) and a low affinity (nucleotide-free) state. This shares features with findings from the HCV NS3 system, but bears a distinction in that NS3 cycles between a low affinity state when nucleotide-bound to a high-affinity state when nucleotide-free. Cluster I is comprised of R685 and T710, whereas cluster II is comprised of R865, N817, T868, and H842. Within these clusters, we distinguish between residues key for loading (R685, T868) and for translocation (T710 and H842), the latter having markedly nucleotide-dependent roles as also observed in the HCV NS3 system. It will be interesting to determine if Mfd operates via a Brownian ratchet that involves translocation in 1 bp steps (like in NS3), and whether and how the binding of RNAP might rectify this ratchet to achieve high processivity on the order of hundreds of base pairs, detected using single-molecule techniques^16,28^. We demonstrate that although there is no biochemical evidence for the TRG helices being directly involved in DNA binding, this structural element in direct communication with ATPase motif VI and the hook is mobile and likely used as a pawl during translocation. Here, we present the first structural evidence for the hypothesized interlocking of nucleotide-responsive elements (the TRG, the hook and relay helices, seen in Fig. 2c, 5) that is driven by movement of ATPase motif VI.

Translocases that move along dsDNA often make more extensive interactions with one DNA strand, termed the tracking strand. In the structure of *S. solfataricus* Snf2, more extensive interactions exist between the tracking strand and motifs I, Ia, Ib, Ic, and IIa^41^, located in the N-terminal lobe (Fig. 2a, c), whereas recent studies of Snf2 bound to a nucleosome suggest tighter interactions with the motifs in the C-terminal lobe^42,43^. It is impossible for us to determine at this stage which of the strand represents the “tracking” strand. This remains an important outstanding question since it underlies strand-specific probing of damage, for whose recognition UvrA, UvrB and Mfd are required^52^.

Our identification of an Mfd family-specific residue important for translocation (H842, located in motif IVa) provides an attractive avenue to pursue in the development of an Mfd inhibitor of high selectivity. Mfd deletion limits the acquisition of antimicrobial resistance in multiple bacteria, including *E. coli*^25^*, Salmonella tyiphimurium*^25^*, Bacillus subtilis*^25^*, Helicobacter pylori*^23^*, Campylobacter jejuni*^22^, and difficult to treat pathogens such as *M. tuberculosis*^25^. Furthermore, in *Bacillus cereus* and *Shigella flexneri*, recent experiments have implicated Mfd in resistance to the nitric oxide response generated by the immune response during infection^24^. Thus, an Mfd inhibitor would provide a novel strategy for antibiotic development. To block function while achieving selectivity, this could target highly variable motif IVa, rather than one of the other, more conserved ATPase motifs. In the NS3 system, the arginine clamp in motif IVa has been shown to be druggable^57^. We note that at the current resolution of our EM reconstruction, establishing whether H842 directly contacts DNA or may exert its effect indirectly by modulating the conformation of the motif IVa loop remains challenging.

Finally, our work provides the first evidence that domain D3 of unknown function might be key for TCR regulation in *E. coli* and related species. While D3 is sandwiched between D1a and D5/D6 in nucleotide-free Mfd, it is repositioned in the presence of DNA and transition state analog to make bridging interactions with D7 (Supplementary Movie 1). Motion of D3 is coupled to a peppermill-like motion of the UvrB homology module relative to the C-terminal region of Mfd (D4D5D6D7), which in turn undergoes local rearrangements to close the D5-D7 interdomain cleft, whereas D4–D7 interactions remain largely unperturbed and the UvrA recruitment surface is unmasked. Our work establishes that these conformational changes are coupled to the active site, primarily through motif VI, but also movements of the TRG, hook and relay helices, which remain unresolved at the residue level and await higher resolution structural and mechanistic analyses.

## Methods

### Cloning, protein expression, and purification

Constructs pMS1-pMS8 were obtained via site-directed mutagenesis of pAD6 (encoding N-terminally His-tagged *E. coli* Mfd^58^) using the method of Edelheit et al.^59^. All constructs were verified by DNA sequencing, and in select cases, purified protein was verified using mass spectrometry. Overexpression and purification was performed according to published protocols by a succession of Ni^2+^-affinity chromatography, His-tag cleavage with Prescission protease, heparin chromatography and gel filtration^59^. For constructs pMS2 and pMS5, HiTrap Q-sepharose column (GE Healthcare) was used instead of heparin affinity as these variants failed to bind to heparin affinity matrix. Size-exclusion chromatography was carried out on a Superdex 200 10/30 Increase column (GE Healthcare) using a buffer consisting of 20 mm Tris pH 8.0, 0.10 m NaCl, 5 mm DTT. Mfd truncations were expressed as N-terminal fusions to polyhistidine tags (Supplementary Table 3), which were cleaved using Prescission protease after the initial immobilized metal affinity chromatography step.

### Triplex displacement assays

To generate the TFO-Triplex, linear ds 70mer DNA (50 nm) was mixed with 5’ Alexa647-labeled 21mer TFO oligo (25 nm) in an opaque tube in triplex reconstitution buffer (10 mm MES pH 5.5, 12.5 mm MgCl_2_) and heated to 57 °C for 15 min. The reaction was then placed at 4 °C and allowed to thermally equilibrate overnight. Protein samples were dialyzed against 10 mm Tris-HCl pH 8, 100 mm NaCl, 10 mm MgCl_2_, 2 mm β-mercaptoethanol. Reactions were assembled by mixing: 10 nm TFO-triplex, 50 nm 44 bp ssDNA trap in reaction buffer (50 mm Tris-HCl pH 8, 10 mm MgCl_2_, 1 mm DTT) and protein (450 nm for constructs used in Fig. 7b–c; 4.5 μμ for the experiments of Supplementary Fig. 8d). Triplex displacement was initiated by addition of 2 mm ATP. Samples were withdrawn at indicated timepoints, stopped by addition of GSM buffer (15% (w/v) glucose, 3% (w/v) SDS, 250 mm MOPS pH 5.5), and loaded on 3–8% Tris-Acetate gels (Invitrogen, Carlsbad, CA), which were pre-run for 60 min at 75 V using Tris-Acetate Running buffer (40 mm Tris-Acetate pH 5.5, 5 mm sodium acetate, 1 mm MgCl_2_) at 4 °C. Samples were electrophoresed for 180 min at 75 V and imaged using a ChemiDoc system (Biorad, Hercules, CA). The disappearance of TFO-Triplex over time was quantified using BioRad ImageLab and plotted using the Graphpad Prism software package.

### Specimen preparation for cryo-EM

Size-exclusion chromatography (Superdex 200 10/30, GE Healthcare) was performed prior to complex reconstitution in a buffer containing 20 mm Tris-HCl pH 8, 100 mm NaCl, 20 mm MgCl_2_, 2 mm TCEP. For complex formation, Mfd was incubated with 21mer dsDNA in a 1:1.5 molar excess in the presence of 2 mm ADP, 10 mm NaF, and 1 mm AlCl_3_. 2.5 µl Mfd-DNA complex at 1.0 mg/mL was applied to UltrAuFoil R1.2/1.3 300 mesh grids (Quantifoil) that were previously plasma-cleaned using a Gatan Solarus (75% argon/25% oxygen atmosphere, 15 W for 7 s), then manually blotted with a Whatman No. 1 filter paper in a cold room with >80% humidity, and plunged into liquid ethane using a manual plunger. The dsDNA used for reconstitution had the following sequence: 5′-ATAGGATACTTACAGCCATCG-3′.

### Cryo-EM data collection

Automated EM image acquisition was performed with Leginon^60^. Data collection was carried out on a Talos Arctica microscope (FEI) operating at 200 kV and equipped with a K2 Summit direct electron detector (Gatan) at a nominal magnification of ×36,000 and a defocus range from 2.0 μm to 4.0 μm giving a pixel size of 1.15 Å at the specimen level. To ameliorate preferential specimen orientation, images were collected using 30° tilts, as previously described^61^. Other than setting the nominal tilt angle during data acquisition, standard procedures were employed. Details are in Supplementary Table 2.

### Cryo-EM image processing

Movies were aligned and dose-weighted using MotionCor2^62^. CTF estimation and particle picking was performed in Warp^63^, which resulted in a stack of 69,721 particles that were imported into Relion (version 3.06)^64^ for 2D classification. One round of 2D classification was performed, and bad particles were excluded. Subsequently, a round of 3D classification, using five classes was run in Relion (Supplementary Fig. 2). The initial model used for 3D classification was generated from a small subset of the data, during data collection using cisTEM^65^. The best class was selected from 3D classification for moving forward. The selected particle stack from Relion contained 19,083 particles and was then input into cryoSPARC for iterative 2D and 3D classification. This cleaned stack containing 9822 particles was then imported into cisTEM and refined using the Auto-Refine procedure, whereby only frequencies up to 80% of Nyquist were included. This resulted in a final resolution of 5.47 Å, assessed by the conventional Fourier Shell Correlation (FSC) criteria. The refined map was sharpened in cisTEM by flattening the amplitude spectrum between 8 Å and the nominal resolution of 5.5 Å. Directional resolution volumes were generated using the 3D FSC tool^61^, whereas the local resolution was calculated using sxlocres.py, which is implemented within the Sparx processing package^66^. Details on model building and refinement are in Supplementary Methods.

### Reporting summary

Further information on research design is available in the Nature Research Reporting Summary linked to this article.

## Supplementary information

Supplementary Information

Description of Additional Supplementary Files

Supplementary Movie 1

Reporting Summary

## Data Availability

The reconstruction of DNA-bound Mfd was deposited in the EMDB under accession code EMD-22146 and model coordinates into the PDB under PDB ID 6XEO. Other data reported here can be obtained upon request from the authors. Source data are provided with this paper.

## Source data

Source Data

## Code availability

Computer code used for calculating sampling distributions and the sampling compensation factor value is available upon request from D.L.
