# Supplementary material for: Molecular determinants for dsDNA translocation by the transcription-repair coupling and evolvability factor Mfd
Source: Nat Commun. 2020 Jul 27;11:3740. doi: 10.1038/s41467-020-17457-1 (PMC7385628; doi:10.1038/s41467-020-17457-1)
Supplement: Supplementary file 1 — Supplementary Information [file 41467_2020_17457_MOESM1_ESM.pdf]

# SUPPLEMENTARY INFORMATION

## Molecular Determinants for dsDNA Translocation by the Transcription-Repair Coupling and Evolvability Factor Mfd

Christiane Brugger, Cheng Zhang, Margaret M. Suhanovsky, David D. Kim, Amy N. Sinclair, Dmitry Lyumkis and Alexandra M. Deaconescu

It includes:

Supplementary Methods

Supplementary Figures 1-9

Supplementary Tables 1-3

## SUPPLEMENTARY METHODS

**Fluorescence anisotropy assays.** A double-stranded 40 bp DNA fragment was generated by annealing two HPLC-purified complimentary oligonucleotides, one of which contains a 5' HEX fluorophore (Integrated DNA Technologies). 150 µL of fluorescently labeled DNA (10 nM) in buffer (20 mM HEPES pH 7.5, 50 mM NaCl, and 2 mM β-mercaptoethanol) was titrated with increasing concentrations of wild-type or variant Mfd. The protein solution and buffer also contained the appropriate nucleotide/nucleotide analog (ADP, ATPγS, or ADP•$AlF_x$) at 2.0 mM. After each addition of Mfd, the reaction was equilibrated for 5 min at 25 °C before measurements were recorded. Fluorescence anisotropy was measured at 555 nm using a Fluoromax-4 spectrofluorometer (Horiba). Background signal corresponding to no protein control was subtracted. Measurements were recorded in triplicate and $K_d$ values were calculated using the Graphpad Prism software package according to the method presented in LiCata et al.[1]

**Preparation of Mfd in the presence of ADP•$AlF_x$.** Mfd was incubated with 2.0 mM ADP and 10.0 mM NaF for 5 minutes before the addition of 2.0 mM $AlCl_3$.

**Circular Dichroism.** Purified Mfd variants were dialyzed overnight into 10 mM potassium phosphate pH 7.6 buffer with 0.5 mM tris(2-carboxyethyl)phosphine (TCEP). Data were collected on a Jasco J-815 CD spectrometer at a protein concentration of 1.5 µM. Measurements were performed at 25 °C in a 2 mm path length cuvette over a wavelength range of 190 - 260 nm with a speed of 50 nm/min. Spectra represent an average of 3 scans corrected for background. For characterization of $Mfd^{D7\text{-}AAA}$ and its variants, the accumulation was increased to 10.

**ATPase assays**. ATPase assays were carried out using an ATP/NADH-coupled ATPase assay at 37°C in a 100 µL reaction volume containing 40 nM wild-type or variant Mfd. Assays were carried out in buffer (40 mM HEPES pH 8.0, 100 mM NaCl, 5 mM KCl, 10 mM $MgCl_2$, 4% glycerol (v/v), 2 mM DTT) containing 0.024 units/µL pyruvate kinase, 0.036 units/µL lactate

dehydrogenase, 5.0 mM phosphoenolpyruvate and 2.0 mM β-nicotinamide adenine dinucleotide (NADH). The reactions were started by the addition of ATP at a final concentration of 4.0 mM and the absorbance at 340 nm was measured every 30 s for 1 h in a Cytation 3 cell-based multi-mode microplate reader (BioTek). Triplicate measurements were performed and the linear decrease in absorbance was used to calculate the rate of NADH oxidation (which is equal to that of ATP hydrolysis) using the molar extinction coefficient for NADH of 6.22 $mM^{-1}$ $cm^{-1}$. DNA stimulation was tested by addition of herring sperm DNA at 0.01 mg/ml.

**Limited proteolysis**. Limited proteolysis was carried out at room temperature in the presence of thermolysin (1:10 molar ratio), trypsin (1:50) and subtilisin (1:50) in a buffer consisting of 20 mM Tris pH8, 150 mM NaCl, 1mM DTT. Incubation times are indicated in Supplementary Fig. 3.

**Model building and refinement**. To build the model of the Mfd-DNA complex, individual protein domains (D1a (1-117), D2 (118-216), D1b (217-353), D3 (354-453), D4 (454-545), D5 (546-780), D6 (781-996) and D7(997-1148)) were extracted from the nucleotide-free Mfd structure (PDB ID: 2EYQ)[2] and fitted individually as rigid bodies into the reconstructed map within UCSF Chimera[3]. Next, the connections between domains that undergo large conformational changes upon dsDNA binding were rebuilt manually in COOT[4] and the 18nt dsDNA model was built in COOT starting from B-form DNA. The final model was refined against the 5.5 Å cryo-EM map in PHENIX[5] using a strategy that includes real-space refinement, morphing and simulated annealing. DNA register could not be established at this resolution and is arbitrary. Figures were prepared in UCSF Chimera[3].

## SUPPLEMENTARY FIGURES

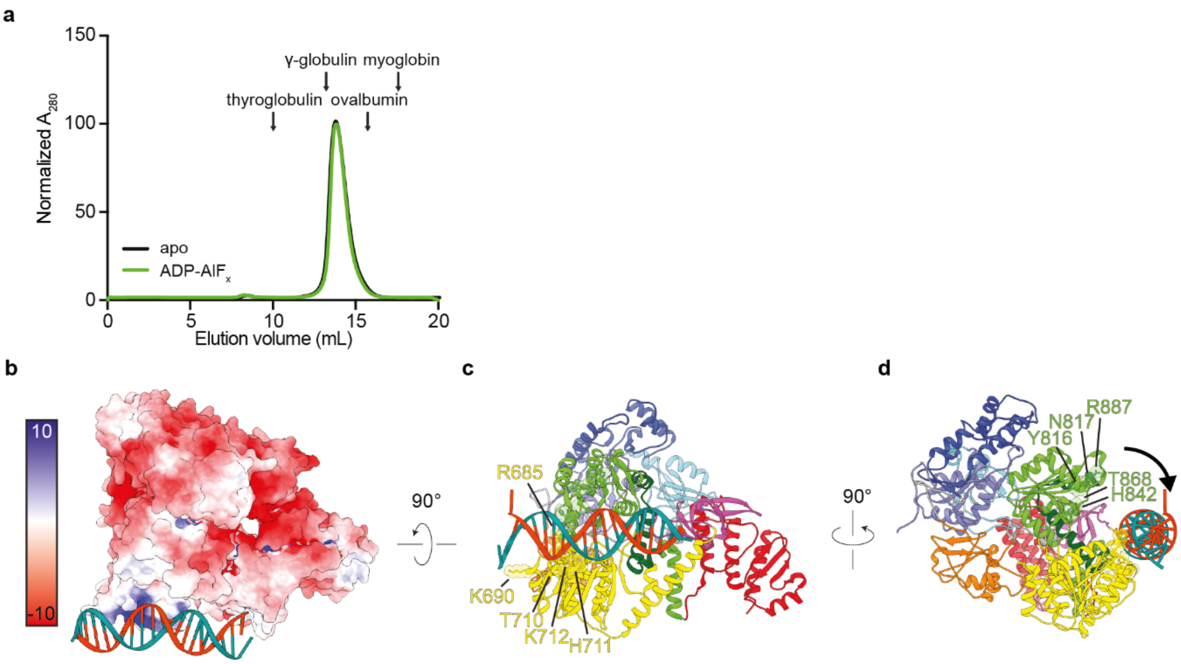


**Supplementary Fig. 1. Mfd is a monomer in all nucleotide states. a)** Superdex 200 size exclusion chromatography elution profiles in the absence of nucleotide (black) and with 2 mM ADP•AlF$_x$ (green) using a mobile phase consisting of 20mM Tris pH 8, 100 mM NaCl, 20 mM MgCl$_2$, 2mM TCEP. The apparent molecular weight determined from the elution profiles (130 kDa) match the theoretical molecular weight of wild-type Mfd (132 kDa). Elution profiles in the presence of ADP and ATP are presented by Deaconescu et al.[29] **b-d)** Model of DNA-bound nucleotide free Mfd based on a superposition with the motor N-lobe of *Sulfolobus solfataricus* Snf2 in complex with dsDNA (PDB ID 1Z63). The electrostatic potential surface and orthogonal views (c,d) highlighting residues presumed to be involved in DNA binding are shown. Color scheme as in Fig. 2a. Electrostatic potential is contoured between -10k$_B$T and 10k$_B$T as indicated by the vertical bar in b, and where k$_B$ represents the Boltzmann constant and T, the temperature in Kelvins.

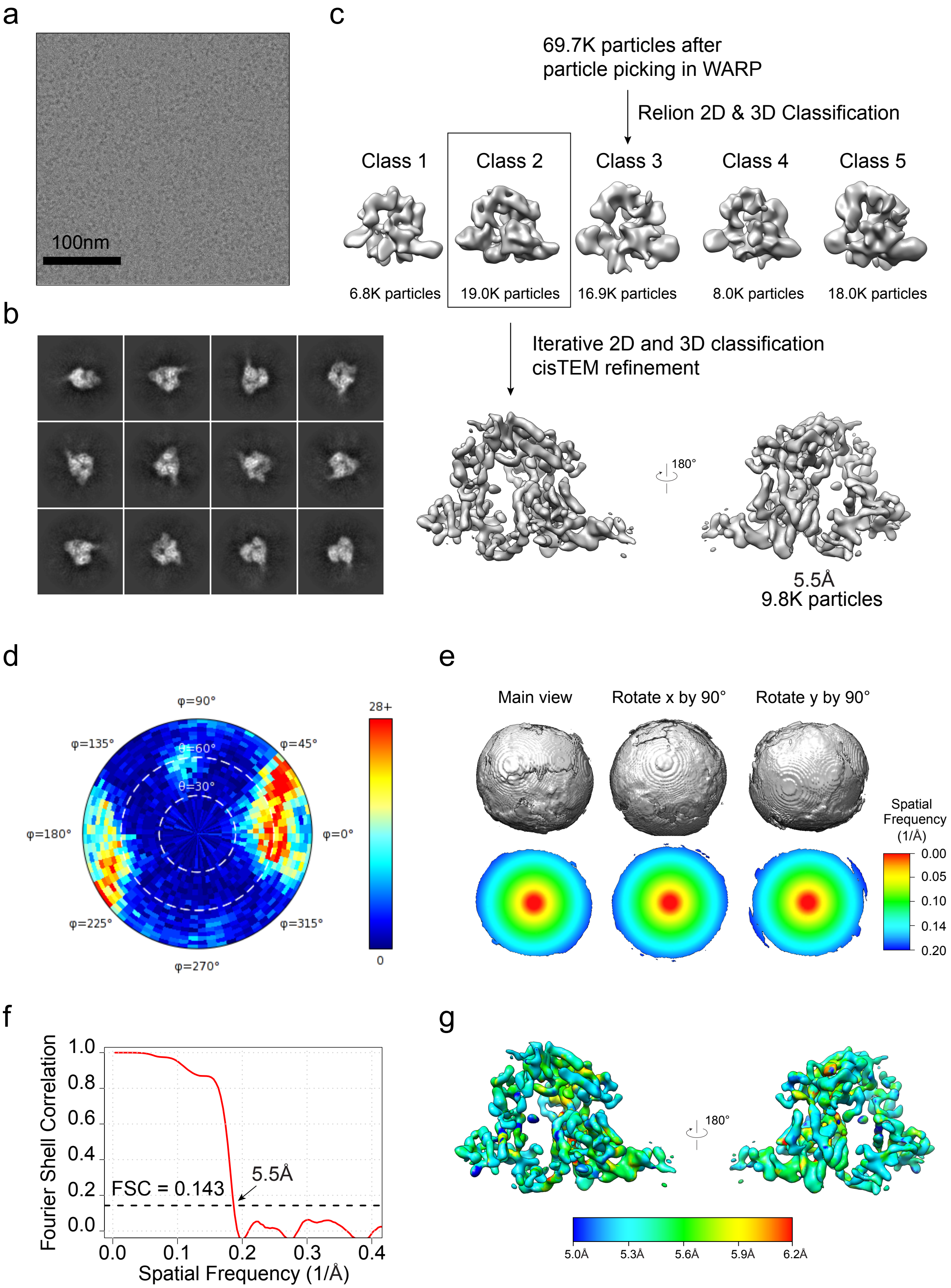
a
100nm
b
c
69.7K particles after
particle picking in WARP
Relion 2D & 3D Classification
Class 1
Class 2
Class 3
Class 4
Class 5
6.8K particles
19.0K particles
16.9K particles
8.0K particles
18.0K particles
Iterative 2D and 3D classification
cisTEM refinement
180°
5.5Å
9.8K particles
d
φ=90°
φ=135°
φ=45°
θ=60°
θ=30°
φ=180°
φ=0°
φ=225°
φ=315°
φ=270°
28+
0
e
Main view
Rotate x by 90°
Rotate y by 90°
Spatial
Frequency
(1/Å)
0.00
0.05
0.10
0.14
0.20
f
Fourier Shell Correlation
1.0
0.8
0.6
0.4
0.2
0.0
FSC = 0.143
5.5Å
0.0
0.1
0.2
0.3
0.4
Spatial Frequency (1/Å)
g
180°
5.0Å
5.3Å
5.6Å
5.9Å
6.2Å

**Supplementary Fig. 2. Cryo-EM data associated with the reconstruction of DNA-bound Mfd.** **a)** A representative cryo-EM micrograph of the Mfd-DNA complex. A total of 652 micrographs were collected from a single EM grid. Scale bar is 100 nm. **b)** Representative 2D class averages of Mfd-DNA complex calculated using cryoSPARC[6] and particles falling into classes that did not show suitable density representative of Mfd were discarded from the data. Scale bar is 100 Å. **c)** Classification strategy and refined maps of the Mfd-DNA complex. **d)** Euler angle distribution plot showing the relative orientation of the particles used in the final 3D reconstruction of the Mfd-DNA complex. The sampling compensation factor (SCF)[7] of the distribution, assuming that all orientations have been correctly assigned, is 0.67. **e)** A 3D FSC volume thresholded at 0.143 is depicted in three orthogonal views. The top three panels show the 3D FSC as an isosurface. The bottom three panels show a slice through the center of the 3D FSC, and the 3D FSC volume is colored by spatial frequency. These are all displayed along the XZ plane (left), XY plane (middle) and YZ plane (right), respectively. **f)** Global Fourier shell correlation (FSC) curve between two half-maps from the data. **g)** Opposing views of the final reconstruction colored by local resolution. Local resolution was calculated using sxlocres.py in Sparx[8].

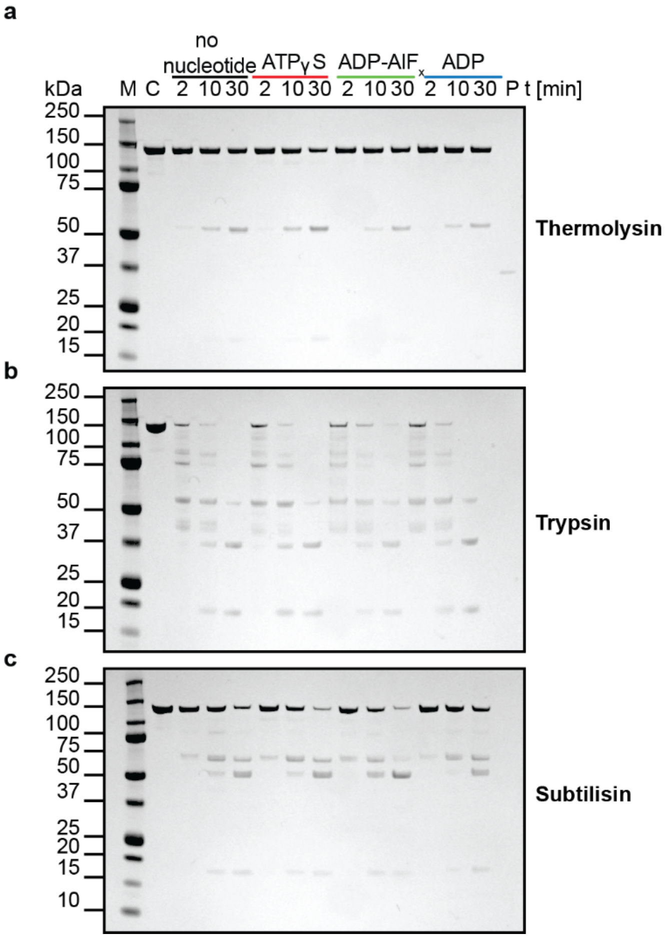


**Supplementary Fig. 3. Limited proteolysis of Mfd. a-c)** SDS-PAGE of Mfd limited proteolysis in the presence/absence of nucleotides and thermolysin (a), trypsin (b) and subtilisin (c). Reactions were incubated for the indicated amount of time. “C” indicates no protease control, and “P” is the protease only control.

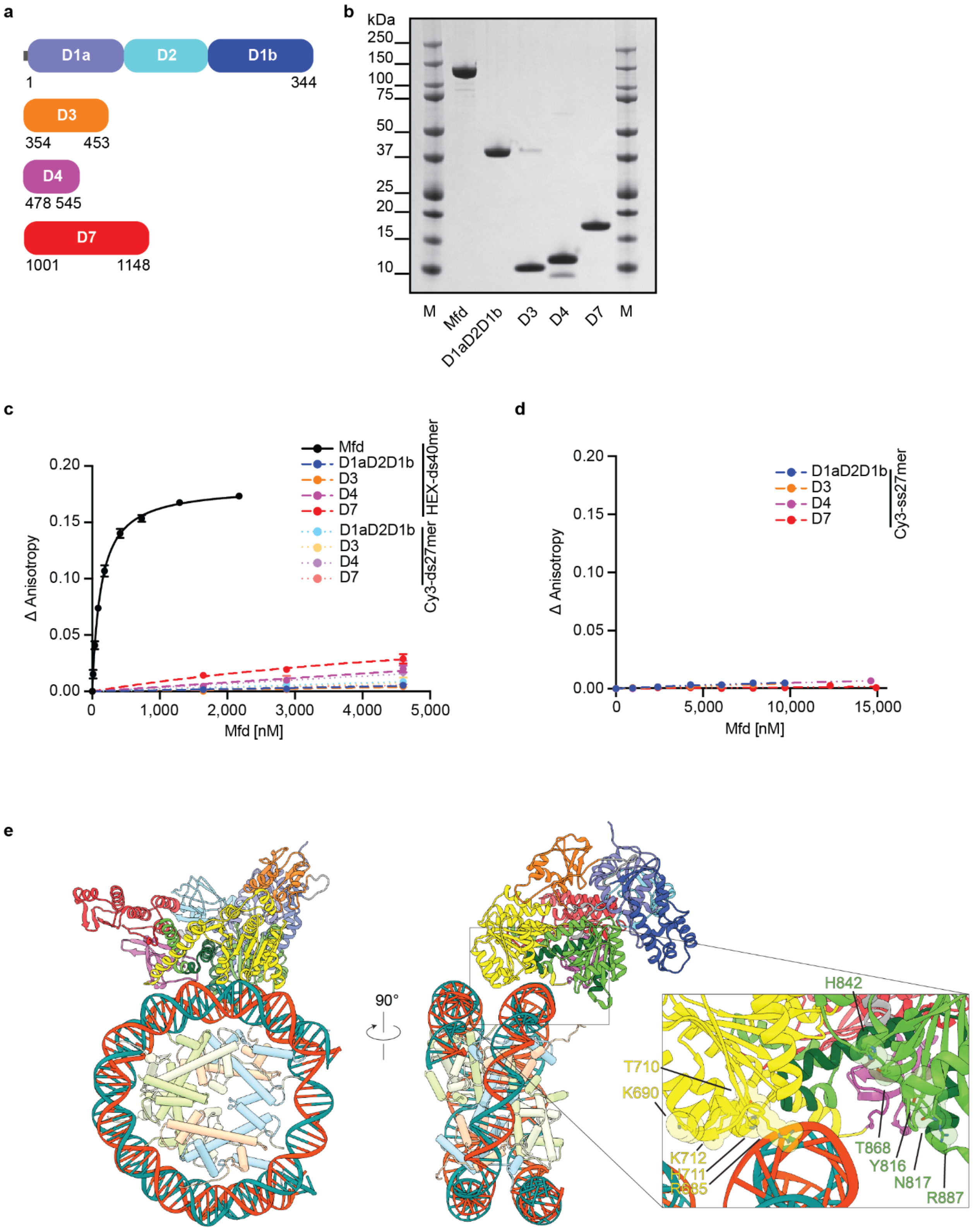


**Supplementary Fig. 4. The Mfd motor core is the primary DNA binding site. a)**Schematic representation of Mfd truncations characterized for DNA binding. **b)** SDS-PAGE analysis of Mfd truncations described in a and used in the experiments of Figure 3c-d. **c)** Fluorescence anisotropy binding curves of full-length Mfd and isolated functional modules. 10nM HEX-labeled 40mer dsDNA in buffer (20 mM HEPES pH 7.5, 50 mM NaCl, 5 mM $MgCl_2$ and 2 mM β-mercaptoethanol)

was titrated with increasing protein concentrations in the absence (dotted line) and presence of 2mM ATPγS (continuous line). After each addition of protein, the reaction was equilibrated for 5 min at 25 °C before measurements were recorded in triplicate. Background signal corresponding to no protein control was subtracted. Error bars represent standard deviation from the mean (N=3) and are often smaller than symbols. Also shown are curves obtained with a shorter Cy3-labeled 27mer dsDNA. **d)** Fluorescence anisotropy binding data obtained for full-length and truncated Mfd and Cy3-labeled 27mer ssDNA. Reactions were performed with 10nM Cy3-labeled 27mer ssDNA in buffer described above and in presence of 2mM ATPγS by titrating increasing protein concentrations. Reactions were equilibrated for 5 min at 25 °C before measurements were taken. Each measurement was performed in triplicate. **e)** Superposition of nucleotide-free *E. coli* Mfd with nucleotide-free Snf2 bound to the nucleosome (PDB ID: 5Z3L) on Mfd D5. Putative residues interacting with DNA are shown as spheres with stick models.

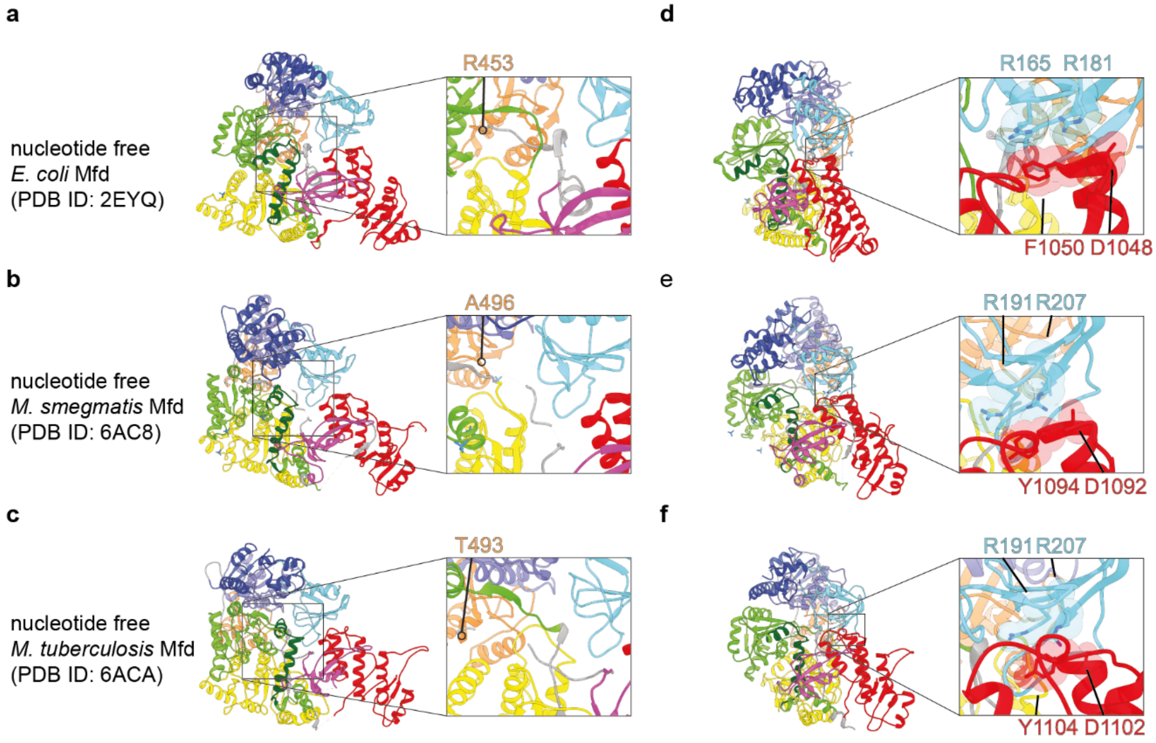


**Supplementary Fig. 5. Structural comparison between mycobacterial and *E. coli* Mfd. a-c)** Top views for structures of Mfd homologs. Insets show the D3-D4 linker D3-D4 with C-terminal residues (R453, A496 and T493) as positional markers. **d-f)** Side views for structures of Mfd homologs with focus on the D2-D7 interface. Amino acids involved in D2-D7 interactions are shown as CPK with stick models and colored according to Mfd domain organization.

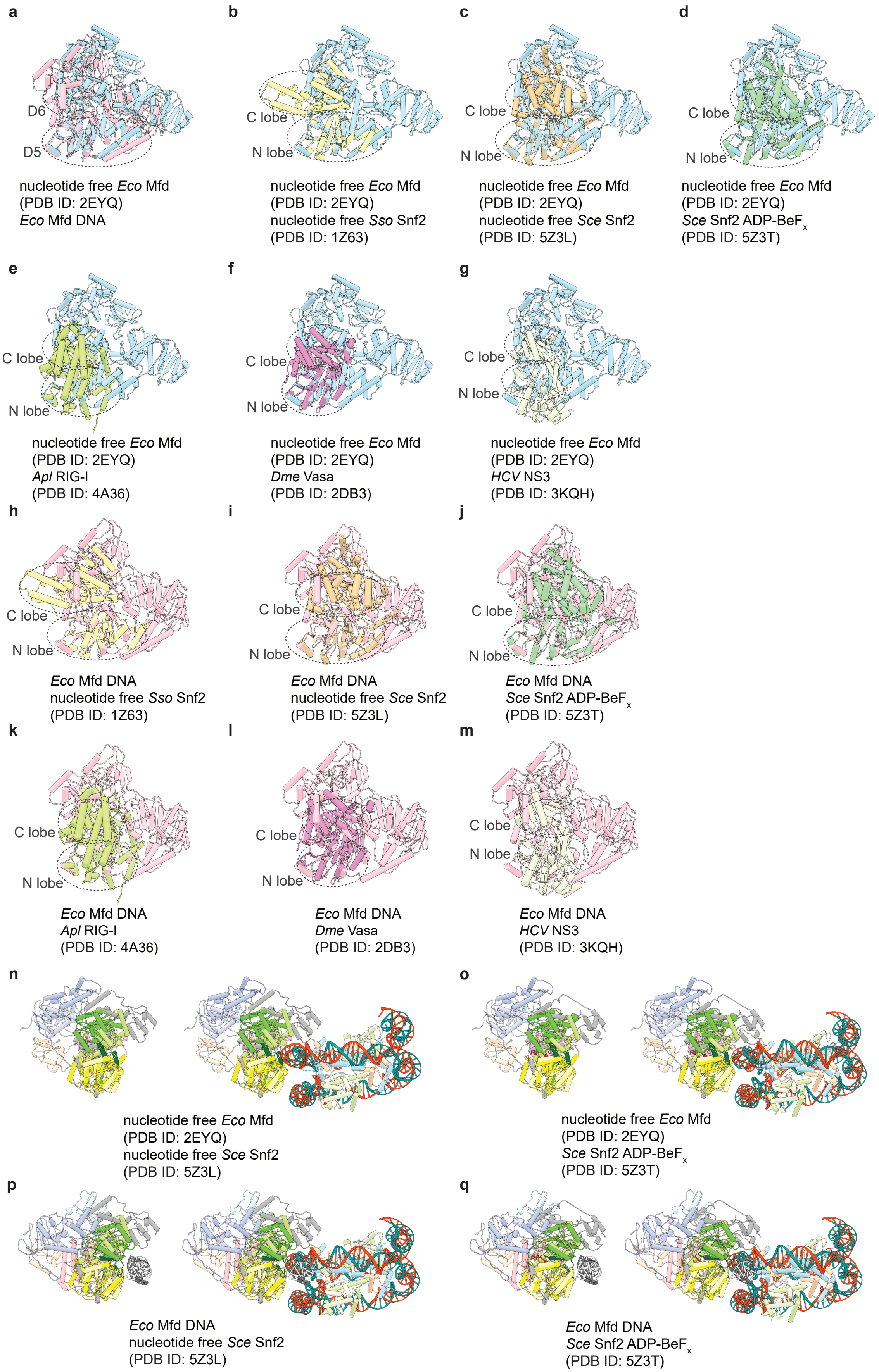
a
D6
D5
nucleotide free Eco Mfd
(PDB ID: 2EYQ)
Eco Mfd DNA
b
C lobe
N lobe
nucleotide free Eco Mfd
(PDB ID: 2EYQ)
nucleotide free Sso Snf2
(PDB ID: 1Z63)
c
C lobe
N lobe
nucleotide free Eco Mfd
(PDB ID: 2EYQ)
nucleotide free Sce Snf2
(PDB ID: 5Z3L)
d
C lobe
N lobe
nucleotide free Eco Mfd
(PDB ID: 2EYQ)
Sce Snf2 ADP-BeF$_x$
(PDB ID: 5Z3T)
e
C lobe
N lobe
nucleotide free Eco Mfd
(PDB ID: 2EYQ)
Apl RIG-I
(PDB ID: 4A36)
f
C lobe
N lobe
nucleotide free Eco Mfd
(PDB ID: 2EYQ)
Dme Vasa
(PDB ID: 2DB3)
g
C lobe
N lobe
nucleotide free Eco Mfd
(PDB ID: 2EYQ)
HCV NS3
(PDB ID: 3KQH)
h
C lobe
N lobe
Eco Mfd DNA
nucleotide free Sso Snf2
(PDB ID: 1Z63)
i
C lobe
N lobe
Eco Mfd DNA
nucleotide free Sce Snf2
(PDB ID: 5Z3L)
j
C lobe
N lobe
Eco Mfd DNA
Sce Snf2 ADP-BeF$_x$
(PDB ID: 5Z3T)
k
C lobe
N lobe
Eco Mfd DNA
Apl RIG-I
(PDB ID: 4A36)
l
C lobe
N lobe
Eco Mfd DNA
Dme Vasa
(PDB ID: 2DB3)
m
C lobe
N lobe
Eco Mfd DNA
HCV NS3
(PDB ID: 3KQH)
n
nucleotide free Eco Mfd
(PDB ID: 2EYQ)
nucleotide free Sce Snf2
(PDB ID: 5Z3L)
o
nucleotide free Eco Mfd
(PDB ID: 2EYQ)
Sce Snf2 ADP-BeF$_x$
(PDB ID: 5Z3T)
p
Eco Mfd DNA
nucleotide free Sce Snf2
(PDB ID: 5Z3L)
q
Eco Mfd DNA
Sce Snf2 ADP-BeF$_x$
(PDB ID: 5Z3T)

**Supplementary Fig. 6. Structural comparison of Mfd with ss/ds DNA translocases. a-g)** Superposition between nucleotide-free *E. coli* Mfd (light blue) and *E. coli* Mfd bound to DNA and ADP•AlF$_x$ (pink, a) *Sulfolobus solfataricus* Snf2 (yellow, b), nucleotide-free *Saccharomyces cerevisiae* Snf2 apo (orange, c), *S. cerevisiae* Snf2 bound to ADP•BeF$_x$ (green, d), *Anas platyrhynchos* RIG-1 (light green, e), *Drosophila melanogaster* Vasa (dark pink, f) and *HCV* NS3 (white, g). All superpositions were restricted to the C$\alpha$ trace of D5. **h-m)** Superpositions between DNA-bound *E. coli* Mfd (pink) and nucleotide-free *S. solfataricus* Snf2 (yellow), *S. cerevisiae* Snf2 in nucleotide-free (orange) and ADP-AlF$_x$-bound (sage) states, *A. platyrynchos* RIG-I (lime), *D. melanogaster* Vasa (hot pink) and HCV NS3 (pale yellow). DNA chains in the Mfd structural model has been removed for simplicity. Superpositions were restricted to the N-lobe of the ATP motor.
**n-q)** Superpositions of nucleotide-free (n,o) and DNA-bound (p,q) Mfd with *S. cerevisiae* Snf2 bound to nucleosomes. The N-lobe of Mfd (D5) is colored yellow, while the C-lobe (D6) is green. The C-lobe of Snf2 is colored sage.

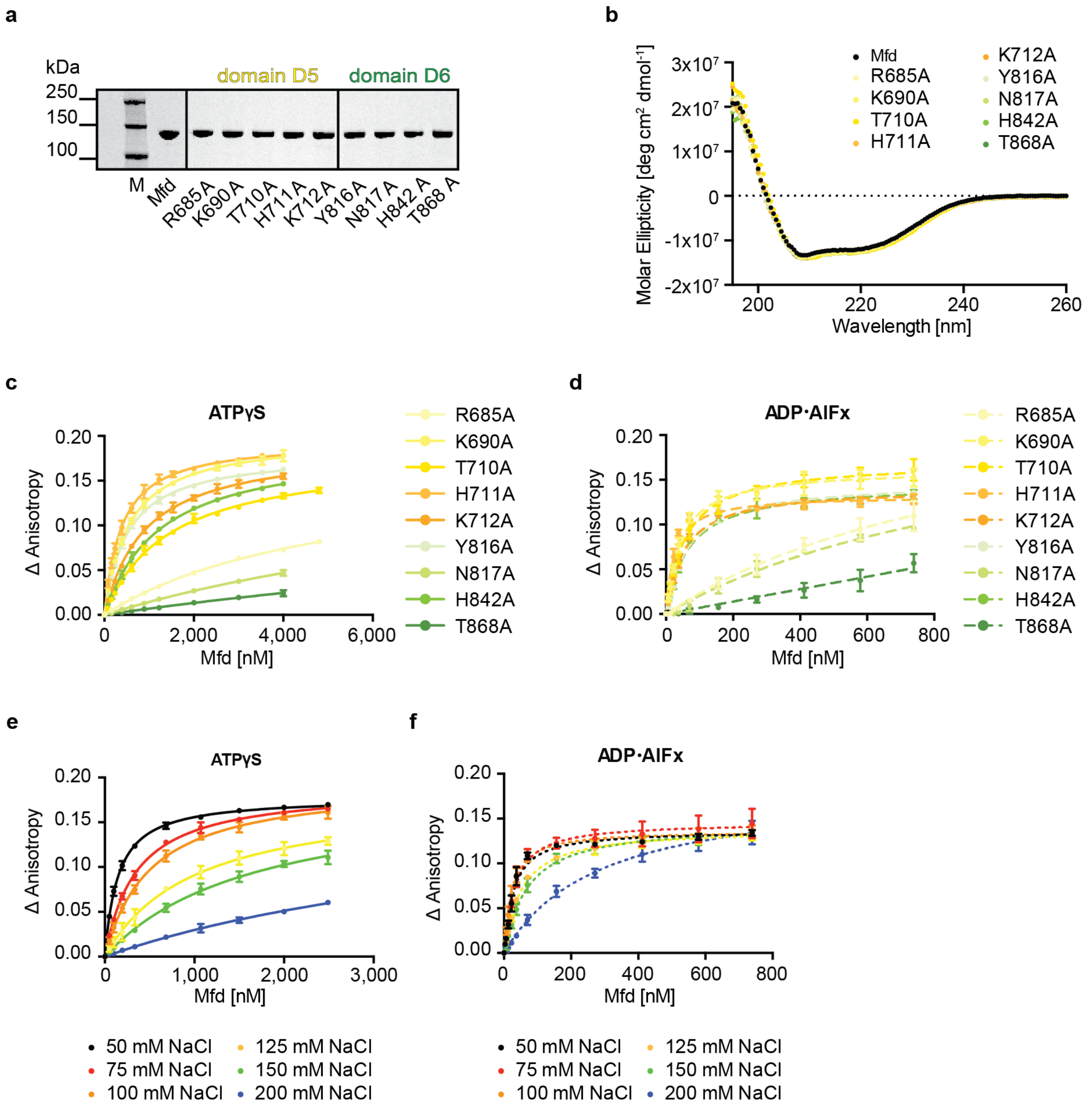


**Supplementary Fig. 7. Functional characterization of Mfd variants utilized in this study. a)** SDS PAGE analysis of full-length Mfd variants used in the experiments described in Figure 6. Constructs were expressed in BL21(DE3) cells and purified by successive immobilized nickel affinity chromatography and tag cleavage, followed by subtractive immobilized nickel affinity, heparin affinity and gel filtration using a previously reported protocol[9]. **b)** Circular dichroism spectra of wild-type and variant Mfd proteins utilized in the experiments of Figure 4. Spectra were obtained as described in Methods with 1.5 µM wild-type or variant Mfd in the absence of nucleotide. **c-d)** DNA binding curves for wild-type and variant Mfd obtained using fluorescence anisotropy on a 40mer HEX-labeled ds DNA in the presence of 2 mM ATPγS (c) and 2 mM ADP•AlF$_x$ (d). Titrations for anisotropy measurements were performed in triplicate and data represent mean values ± SD. **e-f)** DNA binding curves for wild-type Mfd obtained using fluorescence anisotropy on a 40mer HEX-labeled ds DNA in the presence of 2 mM ATPγS (e) and 2 mM ADP•AlF$_x$ (f) Titrations for anisotropy measurements were performed in triplicate at the indicated NaCl concentrations. Data represent mean values (n=3) ± SD.

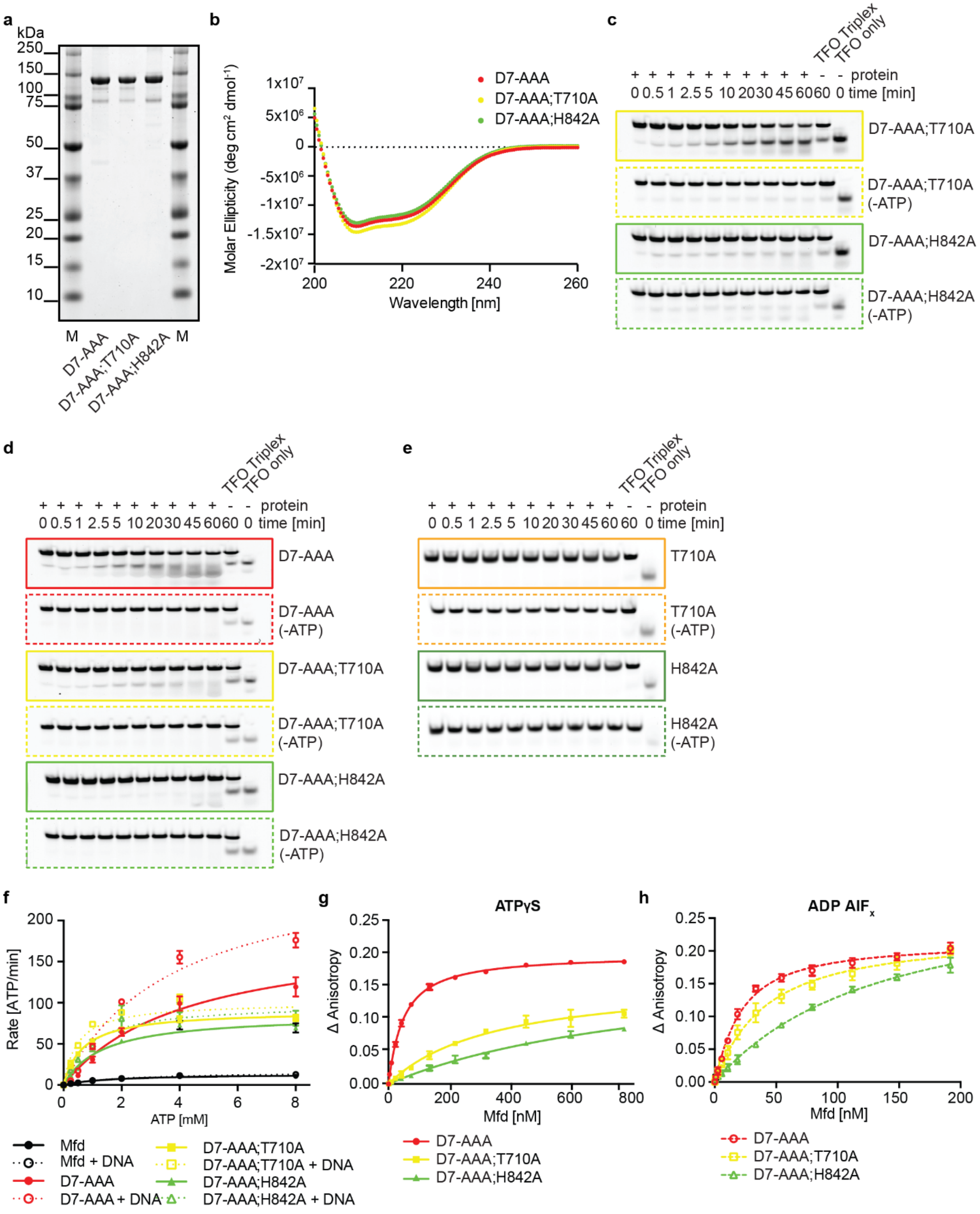


**Supplementary Fig. 8: Mfd variant characterization by triplex displacement assays. a)** SDS PAGE analysis of Mfd$^{D7\text{-}AAA}$, Mfd$^{D7\text{-}AAA;T710A}$ and Mfd$^{D7\text{-}AAA;H842A}$. Constructs were expressed and purified as described in Supplementary Figure 4 and Methods. **b)** Circular dichroism spectra of Mfd$^{D7\text{-}AAA}$, Mfd$^{D7\text{-}AAA;T710A}$ and Mfd$^{D7\text{-}AAA;H842A}$ used in experiments of Figure 6. Spectra were obtained as described in Materials and Methods with 1.5 µM variant Mfd in the absence of nucleotide. **c)** Representative gels of TFO displacement assays carried out with Mfd$^{D7\text{-}AAA}$, Mfd$^{D7\text{-}AAA;T710A}$ and Mfd$^{D7\text{-}AAA;H842A}$ at 450nM concentration. Spontaneous dissociation of TFO-Triplex can

be observed in the TFO-Triplex sample (lane 11) but does not account for the majority of dissociated TFO. Gels for all three replicates are shown as Source Data. **d)** Representative gels of TFO displacement assays carried out with $Mfd^{D7\text{-}AAA}$, $Mfd^{D7\text{-}AAA;T710A}$ and $Mfd^{D7\text{-}AAA;H842A}$ at 4500nM concentration. Gels for all three replicates are shown as Source Data. **e)** Representative gels of TFO displacement assays carried out with Mfd, $Mfd^{T710A}$ and $Mfd^{H842A}$ as in c). Gels for all three replicates are shown as Source Data. **f)** ATPase activity of Mfd variants was measured at 37°C and protein concentrations of 50nM. ATP was added at 0.25/0.5/1/2/4/8mM. Data shown was obtained from three independent experiments, with mean and standard deviations plotted. DNA stimulation was tested by the addition of herring sperm DNA at 0.1mg/mL. **g-h)** DNA binding curves obtained using fluorescence anisotropy of $Mfd^{D7\text{-}AAA}$ variants on a HEX-ds40mer substrate in the presence of 2mM ATPγS (f) or 2mM ADP•$AlF_x$ (g) respectively. Binding assays were performed in triplicate with mean values and standard deviation shown.

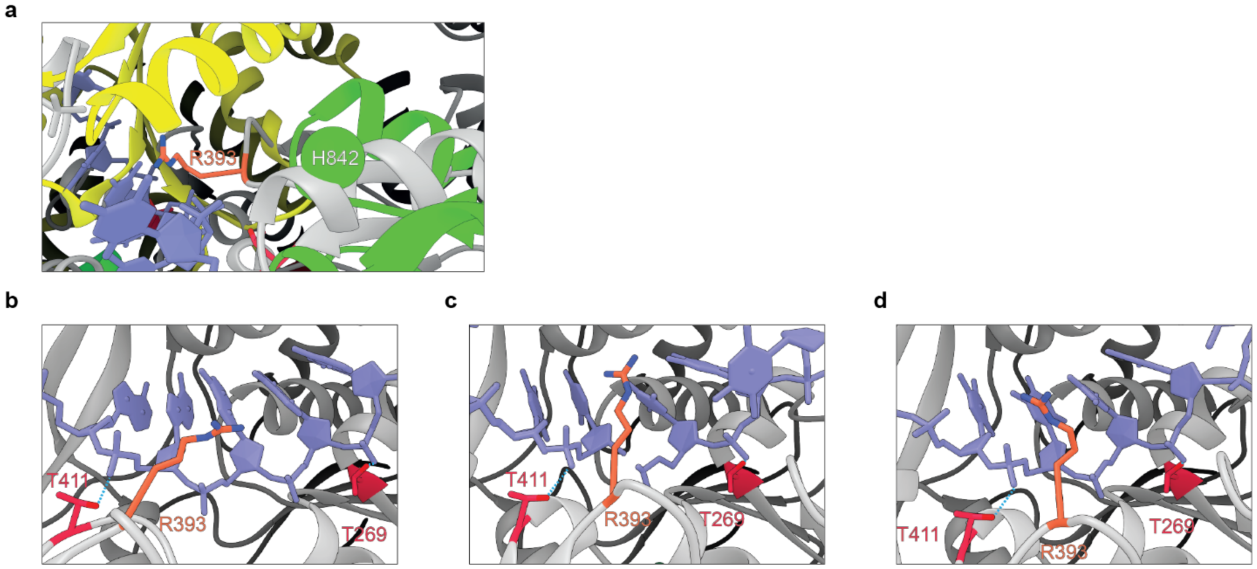


**Supplementary Fig. 9. Mfd and HCV NS3 share a common translocation mechanism. a)** Superposition of Mfd and HCV NS3, highlighting the ratchet translocation mechanism of NS3. Mfd domains are color coded according to Figure 2, NS3 is shown in grey, DNA is shown in purple and important amino acids (Mfd: H842A; NS3: R393) are shown in green and orange respectively. H842 is shown as a C$\alpha$ sphere, while R393 in stick representation. **b-d)** Model of the ratchet translocation mechanism. Three states are shown; (b) free state with no ATP (PDB ID 3KQH); T269 and T411 (shown in red) are located three nucleotides apart; (c) ATP-bound state (PDB ID 3KQU) with T269 and T411, located two nucleotides apart; and (d) the transition analog-bound state (3KQL). Dotted lines indicate key interactions of amino acids and nucleotides.

**Supplementary Table 1. Equilibrium dissociation constants for the Mfd-DNA interaction.** Where appropriate, fitting was not performed (nd) due to very weak binding.

| | ATPγS | | ADP•AlF$_x$ | |
|---|---|---|---|---|
| **Mfd** | **$K_d$ (nM)** | **Standard deviation (nM)** | **$K_d$ (nM)** | **Standard deviation (nM)** |
| Mfd | 151 | 3 | 24 | 2 |
| Mfd$^{R685A}$ | nd | nd | nd | nd |
| Mfd$^{K690A}$ | 538 | 13 | 37 | 3 |
| Mfd$^{T710A}$ | 1428 | 58 | 52 | 4 |
| Mfd$^{H711A}$ | 375 | 12 | 19 | 2 |
| Mfd$^{K712A}$ | 901 | 28 | 46 | 2 |
| Mfd$^{Y816A}$ | 505 | 13 | 58 | 4 |
| Mfd$^{N817A}$ | nd | nd | nd | nd |
| Mfd$^{H842A}$ | 1235 | 39 | 59 | 4 |
| Mfd$^{T868A}$ | nd | nd | nd | nd |
| Mfd$^{R887E}$ | 843 | 35 | 86 | 12 |
| Mfd$^{D7-AAA}$ | 43 | 1 | 16 | 1 |
| Mfd$^{D7-AAA;T710A}$ | 337 | 39 | 35 | 3 |
| Mfd$^{D7-AAA;H842A}$ | 927 | 134 | 127 | 10 |

**Supplementary Table 2. Cryo-EM data collection and processing parameters.**

| Construct | Mfd-DNA Complex |
|---|---|
| **Data Collection and Image Processing** | |
| Microscope | Arctica Talos |
| Voltage (kV) | 200 |
| Camera | Gatan K2 Summit |
| Defocus mean ± std (µm) | 2.7±0.8 |
| Exposure time (s) | 6 |
| Dose rate (e-/pixel/s) | 5.29 |
| Total dose (e- /Å2) | 24 |
| Exposure time per frame (ms) | 100 |
| Number of frames | 60 |
| Pixel size (Å) | 1.15 |
| Number of micrographs | 652 |
| Number of particles (selected) | 69721 |
| Number of particles (in final map) | 9822 |
| Symmetry | C1 |
| Resolution (global) (Å)* | 5.5 |
| Resolution range (homogeneous portion) (Å) | 5.0-6.0 |
| Map sharpening | Spectral flattening between 8Å and 5.5Å |
| **Model Fitting and Refinement** | |
| Initial PDB ID used | 2EYQ |
| Model to map cross-correlation, $CC_{mask}$ | 0.8225 |
| Model composition | |
| Non-hydrogen atoms | 18613 |
| R.m.s. deviations | |
| Bond lengths (Å) | 0.07 |
| Bond angles (°) | 1.133 |
| Validation | |
| Ramachandran plot | |
| Favored (%) | 81.2 |
| Allowed (%) | 18.7 |
| Disallowed (%) | 0.1 |
| Cβ outliers (%) | 0 |
| Rotamer outliers | 0.22 |
| Molprobity score | 2.21 |

*Resolution assessment based on frequency-limited refinement using the 0.143 threshold for resolution analysis

**Supplementary Table 3. Plasmids utilized in this study.**

| Plasmid Name | Description | Reference |
|---|---|---|
| pAD6 | 6His- wild type Mfd | Deaconescu et al (2005)[9] |
| pAS4 | 6His-Mfd$^{R887E}$ | This study. |
| pMS2 | 6His-Mfd$^{R685A}$ | This study. |
| pMS3 | 6His-Mfd$^{K690A}$ | This study. |
| pMS4 | 6His-Mfd$^{H711A}$ | This study. |
| pMS5 | 6His-Mfd$^{K712A}$ | This study. |
| pMS6 | 6His-Mfd$^{Y816A}$ | This study. |
| pMS7 | 6His-Mfd$^{N817A}$ | This study. |
| pMS8 | 6His-Mfd$^{H842A}$ | This study. |
| pMS9 | 6His-Mfd$^{T886A}$ | This study. |
| pMS24 | 6His-Mfd$^{D1aD2D1bA}$ (residues 1-344) | This study. |
| pSKB2-D3 | 6His-Mfd$^{D3}$ (residues 354-453) | This study. |
| pSY4 | 6His-Mfd$^{D4}$ (residues 478-545) | This study. |
| pAD13 | 6His-Mfd$^{D7}$ (residues 1001-1148) | This study. |
| pSY3 | 6His-Mfd$^{T710A}$ | This study. |
| pMfd$^{D7AAA}$ | 6His-Mfd$^{D7\text{-}AAA}$ (E1045A, D1048A and R1049A) | This study. |
| pMfd$^{D7AAA\text{-}T710A}$ | 6His-Mfd$^{D7\text{-}AAA;T710A}$ | This study. |
| pMfd$^{D7AAA\text{-}H842A}$ | 6His-Mfd$^{D7\text{-}AAA;H842A}$ | This study. |

## REFERENCES


1. LiCata, V.J. & Wowor, A.J. Applications of fluorescence anisotropy to the study of protein-DNA interactions. *Methods Cell Biol* **84**, 243-62 (2008).
2. Deaconescu, A.M. et al. Structural basis for bacterial transcription-coupled DNA repair. *Cell* **124**, 507-20 (2006).
3. Pettersen, E.F. et al. UCSF Chimera--a visualization system for exploratory research and analysis. *J Comput Chem* **25**, 1605-12 (2004).
4. Emsley, P. & Cowtan, K. Coot: model-building tools for molecular graphics. *Acta Crystallogr D Biol Crystallogr* **60**, 2126-32 (2004).
5. Adams, P.D. et al. PHENIX: a comprehensive Python-based system for macromolecular structure solution. *Acta Crystallogr D Biol Crystallogr* **66**, 213-21 (2010).
6. Punjani, A., Rubinstein, J.L., Fleet, D.J. & Brubaker, M.A. cryoSPARC: algorithms for rapid unsupervised cryo-EM structure determination. *Nat Methods* **14**, 290-296 (2017).
7. Baldwin, P.R. & Lyumkis, D. Non-Uniformity of Projection Distributions Attenuates Resolution in Cryo-EM. *Prog Biophys Mol Biol* (2019).
8. Hohn, M. et al. SPARX, a new environment for Cryo-EM image processing. *J Struct Biol* **157**, 47-55 (2007).
9. Deaconescu, A.M. & Darst, S.A. Crystallization and preliminary structure determination of Escherichia coli Mfd, the transcription-repair coupling factor. *Acta Crystallogr Sect F Struct Biol Cryst Commun* **61**, 1062-4 (2005).
