# Supplementary material for: Molecular determinants for dsDNA translocation by the transcription-repair coupling and evolvability factor Mfd
Source: Nat Commun. 2020 Jul 27;11:3740. doi: 10.1038/s41467-020-17457-1 (PMC7385628; doi:10.1038/s41467-020-17457-1)
Supplement: Supplementary file 2 — Description of Additional Supplementary Files [file 41467_2020_17457_MOESM2_ESM.pdf]

Description of Additional Supplementary Files

File Name: Supplementary Movie 1
Description: Conformational differences between nucleotide-free Mfd and DNA-bound Mfd
