# Supplementary material for: Molecular determinants for dsDNA translocation by the transcription-repair coupling and evolvability factor Mfd
Source: Nat Commun. 2020 Jul 27;11:3740. doi: 10.1038/s41467-020-17457-1 (PMC7385628; doi:10.1038/s41467-020-17457-1)
Supplement: Supplementary file 4 — Reporting Summary [file 41467_2020_17457_MOESM4_ESM.pdf]

nature research

Corresponding author(s): Alexandra M. Deaconescu

Last updated by author(s): Jun 11, 2020

Reporting Summary

Nature Research wishes to improve the reproducibility of the work that we publish. This form provides structure for consistency and transparency in reporting. For further information on Nature Research policies, see Authors & Referees and the Editorial Policy Checklist.

Statistics

For all statistical analyses, confirm that the following items are present in the figure legend, table legend, main text, or Methods section.

n/a	Confirmed	
☐	☒	The exact sample size (*n*) for each experimental group/condition, given as a discrete number and unit of measurement
☐	☒	A statement on whether measurements were taken from distinct samples or whether the same sample was measured repeatedly
☐	☒	The statistical test(s) used AND whether they are one- or two-sided<br>*Only common tests should be described solely by name; describe more complex techniques in the Methods section.*
☒	☐	A description of all covariates tested
☒	☐	A description of any assumptions or corrections, such as tests of normality and adjustment for multiple comparisons
☐	☒	A full description of the statistical parameters including central tendency (e.g. means) or other basic estimates (e.g. regression coefficient) AND variation (e.g. standard deviation) or associated estimates of uncertainty (e.g. confidence intervals)
☐	☒	For null hypothesis testing, the test statistic (e.g. *F*, *t*, *r*) with confidence intervals, effect sizes, degrees of freedom and *P* value noted<br>*Give P values as exact values whenever suitable.*
☒	☐	For Bayesian analysis, information on the choice of priors and Markov chain Monte Carlo settings
☒	☐	For hierarchical and complex designs, identification of the appropriate level for tests and full reporting of outcomes
☒	☐	Estimates of effect sizes (e.g. Cohen's *d*, Pearson's *r*), indicating how they were calculated

Our web collection on statistics for biologists contains articles on many of the points above.

Software and code

Policy information about availability of computer code

Data collection	Leginon 3.2 (for cryoEM data collection)
Data analysis	Relion 3.0, WARP 1.0.7, cryosparc v2, and cisTEM 1.0.0-beta (for cryoEM data processing), Phenix 1.15.2-3472-000, and Coot 0.892(for model building, and refinement); UCSF Chimera 1.11.2 (build 41380 (for 3D model visualization); Sparx 3.0 (to calculate local resolution); 3dFSC (for evaluating directional resolution) web based version, GraphPad Prism 7

For manuscripts utilizing custom algorithms or software that are central to the research but not yet described in published literature, software must be made available to editors/reviewers. We strongly encourage code deposition in a community repository (e.g. GitHub). See the Nature Research guidelines for submitting code & software for further information.

Data

Policy information about availability of data

All manuscripts must include a data availability statement. This statement should provide the following information, where applicable:

- Accession codes, unique identifiers, or web links for publicly available datasets
- A list of figures that have associated raw data
- A description of any restrictions on data availability

The cryo-EM map for DNA-bound DNA as well as the fitted model have been deposited in the EMDB (accession code EMD-22146) and PDB (PDB I(D 6XEO). A Source Data file is provided with this paper. Any further information can be obtained from the authors upon reasonable request.

Field-specific reporting

Please select the one below that is the best fit for your research. If you are not sure, read the appropriate sections before making your selection.

☒ Life sciences ☐ Behavioural & social sciences ☐ Ecological, evolutionary & environmental sciences

For a reference copy of the document with all sections, see nature.com/documents/nr-reporting-summary-flat.pdf

Life sciences study design

All studies must disclose on these points even when the disclosure is negative.

Sample size	For cryoEM analysis, numbers of particles are reported in Methods.
Data exclusions	For cryoEM analysis, more than 70% of bad particles that would dampen high resolution structural information were discarded after several rounds 2D and 3D classification.
Replication	Replicates are indicated in figure legends when appropriate.
Randomization	No randomization used.
Blinding	No blinding used.

Reporting for specific materials, systems and methods

We require information from authors about some types of materials, experimental systems and methods used in many studies. Here, indicate whether each material, system or method listed is relevant to your study. If you are not sure if a list item applies to your research, read the appropriate section before selecting a response.

Materials & experimental systems

n/a	Involved in the study
☒	☐ Antibodies
☒	☐ Eukaryotic cell lines
☒	☐ Palaeontology
☒	☐ Animals and other organisms
☒	☐ Human research participants
☒	☐ Clinical data

Methods

n/a	Involved in the study
☒	☐ ChIP-seq
☒	☐ Flow cytometry
☒	☐ MRI-based neuroimaging
